# The relationship between body mass and field metabolic rate among individual birds and mammals

**DOI:** 10.1111/1365-2656.12086

**Published:** 2013-05-23

**Authors:** Lawrence N Hudson, Nick J B Isaac, Daniel C Reuman, Daniel Ardia

**Affiliations:** 1 Imperial College LondonSilwood Park, Buckhurst Road, Ascot, Berkshire, SL5 7PY, UK; 2 Centre for Ecology & HydrologyMaclean Building, Benson Lane, Crowmarsh Gifford, Wallingford, Oxfordshire, OX10 8BB, UK; 3 Laboratory of Populations, Rockefeller University1230 York Ave, New York, NY, 10065, USA

**Keywords:** allometry, birds, body mass, body size, daily energy expenditure, doubly labelled water, energetics, field metabolic rate, mammals, metabolic scaling

## Abstract

**Summary:**

1. The power-law dependence of metabolic rate on body mass has major implications at every level of ecological organization. However, the overwhelming majority of studies examining this relationship have used basal or resting metabolic rates, and/or have used data consisting of species-averaged masses and metabolic rates. Field metabolic rates are more ecologically relevant and are probably more directly subject to natural selection than basal rates. Individual rates might be more important than species-average rates in determining the outcome of ecological interactions, and hence selection.
2. We here provide the first comprehensive database of published field metabolic rates and body masses of individual birds and mammals, containing measurements of 1498 animals of 133 species in 28 orders. We used linear mixed-effects models to answer questions about the body mass scaling of metabolic rate and its taxonomic universality/heterogeneity that have become classic areas of controversy. Our statistical approach allows mean scaling exponents and taxonomic heterogeneity in scaling to be analysed in a unified way while simultaneously accounting for nonindependence in the data due to shared evolutionary history of related species.
3. The mean power-law scaling exponents of metabolic rate vs. body mass relationships were 0·71 [95% confidence intervals (CI) 0·625–0·795] for birds and 0·64 (95% CI 0·564–0·716) for mammals. However, these central tendencies obscured meaningful taxonomic heterogeneity in scaling exponents. The primary taxonomic level at which heterogeneity occurred was the order level. Substantial heterogeneity also occurred at the species level, a fact that cannot be revealed by species-averaged data sets used in prior work. Variability in scaling exponents at both order and species levels was comparable to or exceeded the differences 3/4−2/3 = 1/12 and 0·71−0·64.
4. Results are interpreted in the light of a variety of existing theories. In particular, results are consistent with the heat dissipation theory of Speakman & Król (2010) and provided some support for the metabolic levels boundary hypothesis of Glazier (2010).
5. Our analysis provides the first comprehensive empirical analysis of the scaling relationship between field metabolic rate and body mass in individual birds and mammals. Our data set is a valuable contribution to those interested in theories of the allometry of metabolic rates.

The authors provide the first comprehensive empirical analysis of the scaling relationship between field metabolic rate and body mass in individual birds and mammals. The analysis reveals the importance of heterogeneity in the scaling exponent, with consequences for biomass and nutrient flow through communities, and the structure and functioning of whole ecosystems.

## Introduction

Metabolic rate is a fundamental property that dictates daily requirements for individuals and therefore has consequences for biomass and nutrient flow through communities and the structure and functioning of whole ecosystems. Metabolic rate has long been recognized to vary with body mass, M (Kleiber 34; Peters 44; Nagy, Girard & Brown 43), typically expressed as


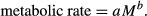
eqn 1

The tendency represented in eqn (1) is enormously important at population (Ernest *et al*. 20; Savage *et al*. 53), community (Cyr & Pace 16; Brose *et al*. 6, Reuman *et al*. 49, 48) and ecosystem (Brown *et al*. 7) levels.

The large majority of past work that has empirically examined the metabolic rate vs. body mass relationship has used basal or resting metabolic rates (BMR or RMR) and/or has used species-averaged estimates of metabolic rate and body mass instead of individual measurements. However, field metabolic rates (FMR) and individual mass and rate phenotypes are more directly ecologically relevant and are probably more directly subject to selection than resting rates and species-average phenotypes, respectively. BMR measures organism metabolism in a calorimeter, but organisms live and interact in the field. Species-average quantities mask variation on which evolution can act, whereas individual analyses capture this variation. Researchers who use the scaling of metabolic rate as a component of their models ultimately seek to understand the behaviour of communities and ecosystems in the field. Individual-level FMR therefore appears to be a more ecologically and evolutionarily relevant measurement to use in the development of ideas about metabolism and its scaling with body size. We therefore compiled the first comprehensive database of measurements of FMR and body mass for individual birds and mammals. We here publish the data and use it to illuminate a series of questions that have long been important topics of debate for BMR/RMR, but that have not been systematically addressed for individual-level field metabolic rates.

For many years, great controversy focussed on whether the value of *b* is closer to 2/3 or 3/4 (reviewed by White & Seymour 63). Scaling of 2/3 is predicted from the ‘surface law’ of metabolism (White & Seymour 63; White 59). The surface law is based on the ratio of volume to surface area, which affects the rates at which heat is produced and lost to the environment. This theory was called into question by empirical data from mammals suggesting that *b* is close to 3/4 (Kleiber 34), leading to the adoption of ‘Kleiber’s law’ of *b* = 3/4, a value that was more recently explained by a theory based on the scaling of circulatory systems and other biological networks (West, Brown & Enquist 58). Heusner’s (30) analysis of 173 individuals of seven mammal species allowed each species to have a different value of *a*; he found that a value of *b* = 2/3 was appropriate for each of his seven species and argued that the value *b* = 3/4 is a statistical artefact of fitting a model that allows a single value of *a*. Feldman & McMahon (22) analysed the same data using a different formulation of the same statistical analysis and found the same values but provided a different interpretation of the results, arguing that *b* = 3/4 and *b* = 2/3 are the appropriate inter- and intraspecific values, respectively, and concluding that *b* = 3/4 is a genuine trend, not an artefact. Further empirical studies have supported *b* = 2/3 (Heusner 30; White & Seymour 62, 63), while others have supported *b* = 3/4 (Feldman & McMahon 22; Savage *et al*. 54; Farrell-Gray & Gotelli 21).

More recent studies focussed on whether a single value of *b* is even appropriate for all clades, and how *b* varies by clade. Such studies often account for nonindependence in the data resulting from shared evolutionary history. White, Phillips & Seymour (60) examined basal rates of fish, amphibians, reptiles, birds and mammals and found significant heterogeneity in *b* among these groups. Capellini, Venditti & Barton (11) investigated mammalian BMR and FMR and found wide variation in *b* among clades, with some having 3/4, some 2/3 and some significantly different from both values. Isaac & Carbone (31) quantified the magnitude of variation in *b* for BMR at different taxonomic levels for a range of animals, finding a mean value of *b* close to 3/4 but large variation at the order level, with 5% of orders lying outside the range 0·54−0·95 and only small amounts of variation at the family and class levels. Analyses of mammalian and avian BMR (McNab 37, 38) have shown that phylogeny and various ecological factors can lead to variation in *b* between clades and found, once these factors had been accounted for, values of *b* = 0·694 for mammals (McNab 37) and b = 0·689 for birds (McNab 38). Glazier’s (25) meta-analysis of metabolic scaling within species, which was based on individual BMR/RMR data, revealed that ontogenetic scaling relationships are variable, often approaching isometry (*b* = 1) and sometimes appearing nonlinear (see also Killen *et al*. 33; Moran & Wells 40; Streicher, Cox & Birchard 56). Individual-level analyses examining both the intra- and interspecific relationships in insects (Riveros & Enquist 51) and terrestrial invertebrates (Ehnes, Rall & Brose 19) have revealed large variation in *b*. Analysis of maximum metabolic rate data from mammals revealed *b*≈7/8 (White & Seymour 63; Gillooly & Allen 23; White *et al*. 64), a value potentially explained by at least two recent competing theories (Glazier, 25, 26–27; Gillooly & Allen 23). These studies illustrate the volume of research that has examined taxonomic heterogeneity of scaling coefficients, *b*, for data that has been on basal or resting rates or has been for species averages.

Of the much smaller collection of empirical studies that have investigated body mass dependence of FMR, all but one have used species-averaged data. These studies have found that *b* is close to 2/3 for birds, close to 3/4 for mammals and close to 8/9 for reptiles (Nagy, Girard & Brown 43; Savage *et al*.’s 54; Anderson & Jetz 2; Nagy 42). Nagy (42) reported that FMR scaling was steeper than BMR scaling for both birds and mammals, although the differences were small and not statistically significant. Anderson & Jetz (2) argued that FMR has an upper limit determined by physiology and a minimum requirement driven by environmental factors. Capellini, Venditti & Barton (11) phylogenetically informed investigation into mammalian FMR found that *b* was not statistically different from 2/3 for their data when considered as a whole but that different orders had confidence intervals that include both, one or none of the values 2/3 and 3/4. Speakman & Król (55) performed both conventional and phylogenetic analyses of species-average FMR of endotherms and found values of *b* not significantly different from *b* = 0·63, the value predicted by their heat dissipation limit theory. The studies surveyed here serve to illustrate the prior work that has examined mass dependence of FMR, albeit for species-averaged data. Riek’s (50) is the only study we are aware of to analyse individual-level FMR. This study argued for the importance of including a random effect of study in statistical models, showing that a linear regression model and a mixed-effects model can give different estimates of *b* = 3/4 and *b* = 2/3 respectively (Riek 50).

A gap in the existing literature is a comprehensive analysis of individual-level FMR data. Within-species scaling of FMR is of interest in its own right, but incorporating this variation into scaling models across species is also likely to be more robust than if it were simply treated as error variance, as in conventional analyses. We compiled the first comprehensive database of measurements of FMR and body mass for individual birds and mammals. We here publish our data and use it to answer four questions. First, what is the magnitude of variation in the exponent *b* among taxa, and at what taxonomic level does variation primarily occur when intraspecific variation is considered alongside variation among species and higher taxa? Second, after accounting for such variation, what are the mean scaling exponents for birds and mammals? Are the mean exponents for each class different from each other and are they closer to 2/3 or 3/4? Third, how does the extent of taxonomic variation in *b* compare to the magnitude of the difference between 2/3 and 3/4, and between the mean exponents for birds and mammals? Finally, what are the implications of our data for existing theory on metabolic rate scaling? These questions have been important in debates centred on species-averaged BMR data, but have not been systematically addressed for individual-level FMR data.

Based on earlier work using species-averaged FMR (Nagy, Girard & Brown 43; Anderson & Jetz 2; Nagy 42; Capellini, Venditti & Barton 11; Speakman & Król 55), we posit the null hypothesis that taxonomic variance in *b* will be statistically meaningful and substantial relative to 3/4−2/3 = 1/12 and relative to the difference between bird and mammal mean slopes. As found by Isaac & Carbone (31) for RMR, we hypothesize that variation will be more important at the order level of taxonomy than the family level. Based on earlier work using individual RMR (Glazier 25), we posit the null hypothesis that species-level variation will also be important and comparable to 1/12. In testing the hypotheses that mean *b* is 2/3 or 3/4, we provide tests of the surface law of metabolism (White & Seymour 63) and of modern theories predicting central tendency values of *b* ≈ 2/3 (Speakman & Król 55) and *b* ≈ 3/4 (West, Brown & Enquist 58; Banavar *et al*. 3; Darveau *et al*. 17; Ginzburg & Damuth 24). In examining taxonomic heterogeneity in *b*, we provide tests of modern theories making predictions about variation (Kozłstrokowski, Konarzewski & Gawelczyk 36; Glazier 25, 26; Savage, Deeds & Fontana 52; Glazier 27; Kolokotrones *et al*. 35; Agutter & Tuszynski 1). More broadly than testing some of the existing theories, this study provides the first comprehensive data set and systematic description of the individual-level FMR-vs.-body mass relationship for birds and mammals.

## Materials and methods

### Database

We obtained all the studies used by Nagy, Girard & Brown (43) together with studies found from our own searches. From these articles, we assembled a database of M measurements (live mass, also known as ‘wet’ mass) and FMR estimates taken using the doubly labelled water technique (described by Butler *et al*. 10). We considered only data resolved to individual level; other criteria for study inclusion are in Appendix S1. In cases where an individual was measured more than once, we computed M and FMR means to get single values for each individual. M was converted to kg and FMR to 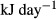. Taxonomy for mammals was from Wilson & Reeder (65) and for birds from Dickinson (18).

### The main set of models

We fitted linear mixed-effects models to the 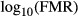-vs.-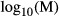 data. Log transformation is standard (e.g. Peters 44) and appropriate (Kerkhoff & Enquist 32) for data of this kind. When equ (1) is fitted to  log-transformed data, *a* is the antilog of the intercept and *b* is the slope. Following the recommendation of Pinheiro & Bates (45, p., log body mass was centred on zero prior to fitting by subtracting the mean of all log body mass measurements from each log body mass measurement. This changes estimates of regression intercepts, but does not affect slopes, which are the subject of this study. All mixed-effects models included fixed effects of taxonomic class (Aves or Mammalia) on both intercept and slope. Class was used as a fixed effect on slope because we are interested in the differences, if any, in slope between birds and mammals. The type I regression models that we used are widely used for analyses of this kind (Nagy 42; Isaac & Carbone 31) and are suitable for our data in part because measurement error in M is very small compared to measurement error in FMR (Butler *et al*. 10; Warton *et al*. 57).

We used taxonomic ranks finer than class to structure hierarchical random effects, following an approach similar to Clarke, Rothery & Isaac (14) and Isaac & Carbone (31). This modelling strategy allowed the variation in slope at each taxonomic rank to be estimated and accounted for the unbalanced nature of the data and nonindependence that results from shared evolutionary history. Random effects at each of the taxonomic ranks of order, family and species were allowed to be either (i) no random effect, (ii) random effect on intercept or (iii) random effect on both slope and intercept, possibly correlated. Thus, there were three options for random effects at three hierarchical levels, giving 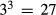 combinations of random effects. Random effects at genus level were not considered because many families are represented by few genera or one genus in our database, so the data were not sufficient to parameterize models with random effects at that level; this modelling choice is consistent with the recommendations of Bolker *et al*. (5, p. 129, Box 5. Some studies presented FMR data for more than one species, and data for some species came from more than one study. To allow for variation in the doubly labelled water protocol (Butler *et al*. 10) and variation in environmental conditions, both of which could affect slope and intercept, all mixed-effects models had a random effect of study on slope and intercept. Models are described using mathematical notation in Appendix S2.

### The main analysis: estimates of slope and heterogeneity in slope

This part of the analysis estimated central tendency values of the exponent *b* for birds and for mammals, the degree of heterogeneity in *b* and the contribution of each taxonomic level to this heterogeneity, answering most of the questions posed in the introduction. We fitted all 27 mixed-effects models to the data. Models were ranked using the Akaike Information Criterion (AIC; Burnham & Anderson 8). The Akaike weight, *w*, was computed for each model. These weights indicate the weight of evidence in favour of each model. We computed model-averaged estimates of fixed-effect slopes for birds and mammals using the Akaike weights and the formulas of Burnham & Anderson (8, p. 152); these estimates can be considered to be central estimates of *b* in eqn (1). Confidence intervals were calculated using the methods of Burnham & Anderson (8, p. 162 and 176). The 95% confidence set of models was computed by progressively summing Akaike weights from highest to lowest until the sum exceeded 0·95 (Burnham & Anderson 8, p. 169).

Random effects are characterized by standard deviations. We computed model-averaged standard deviations of random effects on *b* at the order, family and species level. These values indicated the relative importance of heterogeneity of slope at the taxonomic levels. The absence of a random effect at a given taxonomic level in a model implied a zero standard deviation for that random effect. When model averaging random-effect standard deviations, we therefore used a value of zero for random effects that were not included in models. All 27 models were fitted using restricted maximum likelihood, which gives less-biased random-effect variance estimates than maximum likelihood (Pinheiro & Bates 45, p. 75; Crawley 15, p. 639; Claeskens & Hjort 12, p. 271; Bolker *et al*. 5, p. 128).

### Supporting analyses

We here describe two supporting analyses: one to compare the main set of models with simple models corresponding to the hypothesis that universal relationships between M and FMR exist, and another to examine whether a source of bias described by van de Pol & Wright (46) could have affected results from the main models.

Ordinary linear regression models have historically been used to examine eqn (1) (Nagy 42; Riek 50) and through comparison with the main models allow a test for a universal exponent. We fitted four simple linear regression models, all without random effects. These models had fixed effects of taxonomic class on intercept and had respectively: (i) different slopes for birds and mammals, (ii) the same slope for birds and mammals, (iii) slope 2/3 for both birds and mammals and (iv) slope 3/4 for both birds and mammals.

An assumption of the main set of models is that there are class-specific effects of M on FMR, with random variation around these means at lower taxonomic levels. This is the same as saying that deviations of individual mass from species mean mass have the same effect on FMR as do deviations of species means from family means and deviations of family means from order means. Our main models do not allow for systematic variation in slope at different taxonomic levels; van de Pol & Wright (46) showed that fitting such models when systematic variation is present can produce bias in estimates of random-effect variances. Therefore, to test for the presence of systematic variation, we formulated a second set of 27 mixed-effects models that allowed for such variation, following the framework of van de Pol & Wright (46). The models are described using mathematical notation in Appendix S3. Each model had a random-effect structure comparable to one of the main models. The presence or absence of systematic variation of slope by taxonomic level was detected by the relative AIC rankings of these new models compared to the main models, with low rankings of the new models indicating low potential for bias in results that were based on the main models.

Restricted maximum likelihood fitting could not be used to compare the above models with the main models because it is not appropriate for comparing models with different fixed effects (Pinheiro & Bates 45, p. 76; Crawley 15, p. 636). We used maximum likelihood to (re)fit the main models and to fit both sets of models described above, ranking results by AIC.

### Additional methodological details

All analyses used AIC, which requires a count of model degrees of freedom. It has been suggested that for some applications of mixed-effects models, the number of degrees of freedom contributed by the random effects at a hierarchical level is one per estimated parameter. It has also been suggested that a random-effect level uses degrees of freedom proportional to *m*, where *m* is the number of different categories represented in the data for that random effect (Bolker *et al*. 5, p. 132, Box 3). Claeskens & Hjort (12, p. 270 advise that when the values of specific random effects are important results of an analysis, the latter choice is statistically appropriate, but if only random-effect variances and covariances are needed, degrees of freedom equal to the number of estimated parameters should be used. We set degrees of freedom equal to parameters estimated because our main research goals did not require random-effect levels.

Standard likelihood-based hypothesis tests of random effects are conservative, increasing the risk of type II errors (Bolker *et al*. 5, p. 132, Box 3); in other words, using such tests will tend to select those models that exclude random effects that should be included. Standard AIC-based methods also favour smaller models with random effects omitted (Greven & Kneib 28). Methods for correcting for this bias are still an ongoing topic of statistical research and have not been settled (Greven & Kneib 28). We used the standard AIC-based methods while being aware of the bias: the importance of each random effect is likely to be an underestimate, such that our results are conservative with respect to identifying the taxonomic levels at which *b* varies.

All analyses were conducted using R 2.13.0 (R Development Core Team 47). All mixed-effects models were fitted using the lme4 package (Bates, Mächler & Bolker 4).

## Results

**Figure 1:**
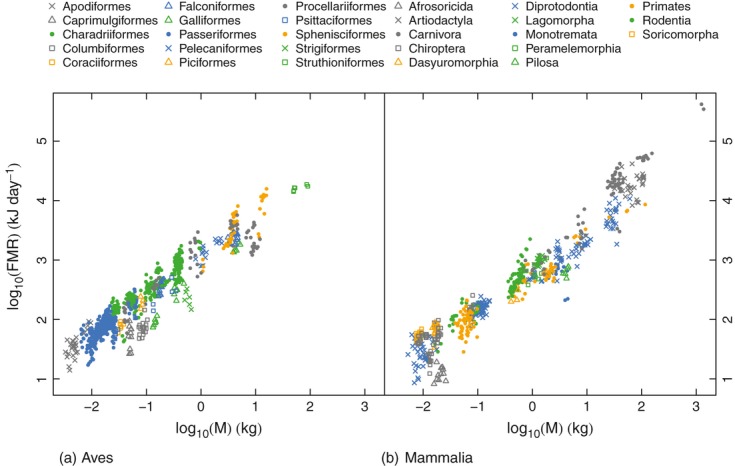
Field metabolic rates (FMR) against M for (a) birds and (b) mammals. Each point is for an individual animal; some points are the average of more than one measurement.

The database contains 1498 individuals from 76 species of birds and 57 species of mammals; 28 orders are represented. Body masses span nearly six orders of magnitude, from 3·3 g for *Archilochus alexandri* (black-chinned hummingbird) to 1370 kg for *Odobenus rosmarus* (walrus). Most individuals in the database were measured once (90·12%) or twice (8·34%). The data are shown in Fig. 1 and provided in full with references in Appendices S5 and S6.

Results for the restricted maximum likelihood fitting of the main set of 27 mixed-effects models are shown ranked by AIC in Table 1. No model had Akaike weight, *w*, >0·9, indicating that none of the models was conclusively the best (Burnham & Anderson 8). The 95% confidence set of models is made up of six models, all of which included random effects for slope at the species level and many of which had random effects for slope at the order level. This provides our first result: data strongly support the presence of heterogeneity in the relationship between individual body mass and field metabolic rate, and heterogeneity is concentrated at the order and species levels. In other words, scaling exponents differ among taxonomic groups across a range, and order- and species-level taxonomic classifications are particularly important for these differences, more so than family-level classifications.

**Table 1:** The 27 mixed-effects models fitted by restricted maximum likelihood and ranked by AIC. Models could have random effects on either intercept (I) or slope and intercept (S & I), at each of the taxonomic levels order, family and species. *K* is the number of model parameters. 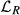 is the restricted maximum likelihood. ΔAIC is the difference between the best model’s AIC and the AIC of the model in question. *w* is the Akaike weight; ∑*w* = 1

Rank	Random effects				*K*	log( 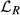 )	AIC	ΔAIC	*w*	∑(*w*)
	Order	Family	Species							
1	S & I	I	S & I	15	1011·832	−1993·665	0·000	0·4003	0·4003
2	I	I	S & I	13	1009·811	−1993·622	0·043	0·3919	0·7922
3	I	S & I	S & I	15	1010·024	−1990·049	3·616	0·0657	0·8578
4	S & I	S & I	S & I	17	1011·845	−1989·689	3·975	0·0549	0·9127
5	S & I		S & I	14	1008·400	−1988·801	4·864	0·0352	0·9479
6	I		S & I	12	1005·875	−1987·751	5·914	0·0208	0·9687
7	S & I	I	I	13	1006·683	−1987·366	6·299	0·0172	0·9858
8	S & I		I	12	1005·082	−1986·163	7·501	0·0094	0·9952
9	S & I	S & I	I	15	1006·684	−1983·368	10·297	0·0023	0·9976
10	I	S & I	I	13	1003·915	−1981·831	11·834	0·0011	0·9986
11	I	I	I	11	1001·898	−1981·796	11·869	0·0011	0·9997
12	I		I	10	999·302	−1978·604	15·061	0·0002	0·9999
13		I	S & I	12	1000·279	−1976·559	17·106	<0·0001	1·0000
14		S & I	S & I	14	1000·658	−1973·316	20·349	<0·0001	1·0000
15		S & I	I	12	994·610	−1965·221	28·444	<0·0001	1·0000
16		I	I	10	990·907	−1961·814	31·850	<0·0001	1·0000
17	S & I	I		12	987·596	−1951·192	42·472	<0·0001	1·0000
18	S & I	S & I		14	988·379	−1948·758	44·907	<0·0001	1·0000
19	I	S & I		12	985·285	−1946·570	47·095	<0·0001	1·0000
20	I	I		10	982·851	−1945·701	47·964	<0·0001	1·0000
21	S & I			11	983·157	−1944·314	49·351	<0·0001	1·0000
22			S & I	11	979·395	−1936·790	56·874	<0·0001	1·0000
23	I			9	975·638	−1933·275	60·389	<0·0001	1·0000
24		S & I		11	976·514	−1931·028	62·637	<0·0001	1·0000
25		I		9	974·104	−1930·208	63·456	<0·0001	1·0000
26			I	9	973·479	−1928·957	64·708	<0·0001	1·0000
27				8	928·908	−1841·815	151·849	<0·0001	1·0000

Model-averaged estimates of the variances of the random effects of each taxonomic level on slopes (Table 2) support the above result: taxonomic slope heterogeneity is greatest at the order level, with a slightly smaller but still important component of heterogeneity at the species level. Standard deviations of order and species random effects were comparable to or exceeded the difference 3/4−2/3 = 1/12 = 0·0833 (Table 2). In other words, theoretically based arguments about whether average scaling exponents are closer to 2/3 or 3/4 may be of secondary importance given that taxonomic variation in scaling exponents easily exceeds the difference between these quantities; explaining taxonomic variation in scaling exponents may be more important.

**Table 2:** Parameter estimates for the main set of mixed-effects models fitted by restricted maximum likelihood. Estimates are provided for the six models that make up the 95% confidence set and averaged over all 27 models. We derived model-averaged random effects standard deviations by taking the square root of model-averaged variances, which were calculated using the approach of Burnham & Anderson (8, p 162)

		Fixed-effects slopes (95% CI)		Random effects SD			
Rank	*w*	Aves	Mammalia	Order	Family	Species	Study
1	0·4003	0·725 (0·630,0·819)	0·635 (0·541,0·729)	0·11924	0	0·05435	0·08160
2	0·3919	0·694 (0·634,0·753)	0·646 (0·592,0·700)	0	0	0·06393	0·08601
3	0·0657	0·692 (0·631,0·753)	0·644 (0·589,0·699)	0	0·03570	0·06065	0·08768
4	0·0549	0·725 (0·630,0·819)	0·635 (0·542,0·728)	0·11864	0·00473	0·05431	0·08244
5	0·0352	0·733 (0·635,0·830)	0·632 (0·535,0·728)	0·12659	0	0·06429	0·07579
6	0·0208	0·693 (0·631,0·755)	0·637 (0·586,0·688)	0	0	0·08020	0·09323
Averaged		0·710 (0·625,0·795)	0·640 (0·564,0·716)	0·08709	0·00962	0·05888	0·08373

Although the random effect for species is present in all the best models, the magnitude of variation in *b* at this level is slightly smaller than the variation among orders. Slope heterogeneity at the species level is more important than at the family level. Heterogeneity at the species level can, of course, only be detected with individual-level data of the kind we have gathered. The study random effect also showed great heterogeneity in slope, with standard deviation exceeding 1/12 (Table 2). In other words, theoretically based arguments about whether average scaling exponents are closer to 2/3 or 3/4 are also substantially confounded by methodological differences among studies. Because our analysis generally supports the presence of important random effects, correcting the bias towards models with simpler random-effect structure in AIC-based approaches (‘Additional methodological details’) would only accentuate our results, if such a correction was available.

Estimates of fixed-effect slopes are shown in Table 2, providing our next result: that the central tendency relationship between individual 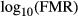 and 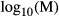 has slope 0·710 (95% CI 0·625–0·795) for birds and 0·640 (95% CI 0·564–0·716) for mammals. The slope 3/4 is excluded for mammals but included for birds; confidence intervals for both classes include 2/3.

Because taxonomic variability in slope (standard deviations of random effects on slope) exceeded or was comparable to the difference 3/4−2/3 = 1/12 at order and species level (Table 2), even given a mean slope close to 2/3 (e.g. for mammals), slopes measured for individual orders or species will often be expected to equal or exceed 3/4. Both mean-slope estimates have wide confidence intervals, and the point estimates for each class are within the confidence intervals for the other class, suggesting no meaningful difference in average slope between birds and mammals. Standard deviations of order, species and study random effects on slope exceeded or were comparable to the difference 0·710−0·640 between bird and mammal mean slopes (Table 2), so many bird orders may have scaling exponent lower than many mammal orders even though the point estimate of the central tendency exponent for birds is higher than that for mammals. This means, in particular, that it may be more important to focus on understanding variation in scaling exponents among orders within birds and mammals than it is to focus on the difference between bird and mammal central tendency exponents.

We examined the goodness-of-fit of our most complex ‘global model’, the mixed-effects model with random effects on both slope and intercept of order, family and species. Residual analyses for this model are in Figs S1–S4. To further demonstrate the fit of this model to the data, we present its predictions for birds and mammals, by order, in Figs S5–S6.

We compared fits of our main models to fits of models that allowed for systematic variation in slope by taxonomic levels and with simple linear regression models (‘Supporting analyses’). The 95% confidence set (Table S1) is entirely from the main models, revealing that the main models were a much better fit. We could not produce estimates of random-effect variances averaged across all the models of Materials and methods because these models could not be compared using restricted maximum likelihood fitting due to heterogeneous fixed effects, and because maximum likelihood produces biased random-effects variance estimates. The choice to produce model-averaged results over the main models (Tables 1 and 2) was appropriate because the main models were much better supported. Lastly, we compared the effect of using the small-sample-corrected AIC, 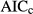, instead of AIC; results were substantially the same (Table S2).

## Discussion

This study is the most comprehensive analysis to date of the body mass scaling of individual FMR. Our analysis accounts for nonindependence in the data arising from shared evolutionary history and looks at both mean scaling exponents and taxonomic heterogeneity in scaling exponents in a unified framework. Results confirmed our hypotheses that (i) taxonomic heterogeneity in scaling exponent is statistically meaningful (i.e. strongly supported by our AIC results) and substantial relative to the difference 3/4−2/3 and the difference between the mean slopes for birds (0·71) and mammals (0·64); and (ii) variation is most important at the order and species levels of taxonomy. Hence, taxonomic variation in scaling exponents easily exceeds differences among various theoretical predictions for average scaling exponent, seeming to diminish in importance debates about what is the ‘correct’ average scaling exponent, and what are the reasons for it, relative to the importance of explaining taxonomic variation in scaling exponents. In the following sections, we compare our average exponent results with the predictions of several theories, as well as, and more importantly in our view, comparing our results about variation in exponents to theory. We also examine the issue of curvature in plots of log metabolic rate vs. log body mass, because it pertains to the comparisons with theory. Results support the heat dissipation limit theory of Speakman & Król (55) and the metabolic levels boundary hypothesis of Glazier (27) more so than other theories.

### Mean slopes and comparison with theory

Our results were consistent with 3/4 as a central exponent value for birds but not for mammals; results were consistent with 2/3 for both birds and mammals. These findings contradict previous studies that examined species-average mammalian FMR data and found *b* close to 3/4 (Nagy, Girard & Brown 43; Savage *et al*. 54; Nagy 42). Our statistical approach refines the approaches of these earlier studies; improved methods may explain the differences between our results and earlier results, as may our use of individual data. Our result for mammals is similar to that of Capellini, Venditti & Barton (11), who found *b*=0·697 (95% CI 0·653–0·741) for species-average mammalian FMR.

Of the many theories that propose mean values of *b*, the heat dissipation limit theory of Speakman & Król (55) seems the most directly relevant to our study because it is formulated explicitly for FMR of endotherms. The theory posits that in times when food supply is not limiting, metabolic rates are limited by the capacity to dissipate heat. Speakman & Król (55) compiled a species-level data set and found *b* = 0·647 for mammals and *b* = 0·658 for birds. Both values were not significantly different from the value *b*=0·63 predicted by their theory. Our results for birds of *b*=0·710 (95% CI 0·625–0·795) and for mammals of *b*=0·640 (95% CI 0·564–0·716) both have confidence intervals that encompass 0·63 despite our use of data containing a different subset of species; an individual-level analysis; and a different statistical approach. Our mean-slope results do not support theories that predict *b* ≈ 3/4, at least not for mammals. These include supply-network theories (West, Brown & Enquist 58; Banavar *et al*. 3), the theory of Darveau *et al*. (17), which combines multiple physiological limitations of metabolic rate, and that of Ginzburg & Damuth (24), which considers organisms to be four dimensional (three dimensions of space and one of time), while dissipating heat through only three dimensions (two of space and one of time).

### Curvature

Recent work examining species-averaged data detected significant convex curvature in log RMR vs. log body mass scatter plots for mammals (Kolokotrones *et al*. 35; see also Hayssen & Lacy 29); discrepancies among prior empirical studies of the scaling of mammalian RMR were explained as a result of curvature, with studies focusing on smaller body masses reporting slopes close to 2/3 and studies focusing on larger masses reporting slopes close to 3/4. Our FMR data for mammals also appear to show convex curvature (Fig. 1; Fig. S7 for significance), but a focus on smaller body masses cannot explain the fact that our mean slope for mammals was close to 2/3 because we did not focus on smaller body masses: the range of masses we used was similar to that of the large collections of Kolokotrones *et al*. (35). Savage, Deeds & Fontana (52) and Kolokotrones *et al*. (35) offered refinements to the supply-network theory to explain observed curvature in their RMR plots. The theory of quantum metabolism also predicts curvature (Agutter & Tuszynski 1). However, heat dissipation limit theory (Speakman & Król 55) provides an alternative explanation for apparent curvature that seems better supported by the FMR data presented here. This theory suggests that the greater thermal conductivity of water compared to air leads to a greater capacity to dissipate heat and therefore a higher FMR in aquatic animals. Data for aquatic mammals should therefore exhibit the same slope but a higher intercept than terrestrial mammals on log FMR vs. log body mass plots (Speakman & Król 55). Of the 56 mammalian individuals in our data set that are aquatic, 51 have a body mass >10 kg (Fig. S7). We tested the hypothesis that the apparent curvature in our mammalian data results from the presence of many large-bodied aquatic animals by fitting three models to our mammalian data: a linear model, a quadratic model and a linear model with different intercepts for aquatic and nonaquatic species. The latter model gave the best fit and had higher intercept for aquatic mammals than for nonaquatic ones (Fig. S7), supporting the heat dissipation theory explanation for apparent curvature. Curvature is not real, in the sense that it can be explained best by linear models with regression line elevations varying by group in a way consistent with the heat dissipation limit theory.

As the theory of West, Brown & Enquist (1997) was originally billed as a universal theory, one may expect its generalizations (Savage, Deeds & Fontana 52; Kolokotrones *et al*. 35) to also be universally applicable and to predict curvature for birds as well as mammals. Our avian data do not appear curved (Fig. 1; Fig. S7 for statistical tests). While potentially inconsistent with the models of Savage, Deeds & Fontana (52) and Kolokotrones *et al*. (35), this is consistent with the heat dissipation theory because aquatic birds are not so predominantly large as to cause curvature in scatter plots by having elevated FMR. We again fitted a linear model, a quadratic model and a linear model with different intercepts for aquatic and nonaquatic birds, repeating this for a variety of ways of categorizing birds as aquatic/nonaquatic (Fig. S7). In all cases, the two-intercept model was the best fit, and the intercept for aquatic birds was higher than that for nonaquatic. These arguments do not disqualify the theories of Savage, Deeds & Fontana (52) and Kolokotrones *et al*. (35) but they do suggest that researchers could usefully examine what predictions those theories make for heterogeneity of curvature across major taxa.

Other empirical studies have found no or limited evidence of curvature in some data sets (Capellini, Venditti & Barton 11; Isaac & Carbone 31), and a recent study suggested curvature is specific only to certain mammalian clades (Müller *et al*. 41). If some groups within each data set, such as aquatic representatives in mammalian and bird data sets, are more able to dissipate heat than others, one may expect heterogeneous curvature results for different data sets according to whether better heat dissipators are larger or smaller than other organisms considered in the particular data set, or distributed evenly across body masses. Ehnes, Rall & Brose (19) found curvature in basal rate data for soil invertebrates, and some studies have shown that intraspecific scaling can be nonlinear for various ectotherms (Glazier 25; Killen *et al*. 33; Moran & Wells 40; Streicher, Cox & Birchard 56); these results are interesting but not directly relevant to heat dissipation theory, which applies to endotherms.

### Heterogeneity in slopes and comparison with theory

In agreement with previous studies (e.g. Capellini, Venditti & Barton 11), we found variability in *b*. Isaac & Carbone (31) showed that for species-averaged basal rates, the mean slope 3/4 was well supported, but that taxonomic variability around that mean was sufficiently great that, for instance, ‘extreme’ values outside the range 0·5–1 should not be unexpected even for whole orders. Our conclusions are analogous: our order-level random-effect standard deviation was 0·0871, compared with 0·105 for RMR across metazoa in Isaac & Carbone (31). This means that, for individual FMR data, our model predicts that 5% of bird orders will have slopes outside the range 0·54–0·88 and 5% of mammal orders should have slopes outside the range 0·47–0·81. These values are quantiles for normal distributions with means 0·71 and 0·64 and standard deviations 0·0871.

Riek’s (50) is the only other analysis of individual FMR data we are aware of, but that study is limited to arguing for the importance of including a random effect of study in models (which we did). We find it counter-intuitive to model only the random effects of study while ignoring the pseudo-replication resulting from shared evolutionary history. Our results show that taxonomic heterogeneity of slope, particularly at the order level, is at least as important as heterogeneity related to study effects (Table 2).

**Figure 2:**
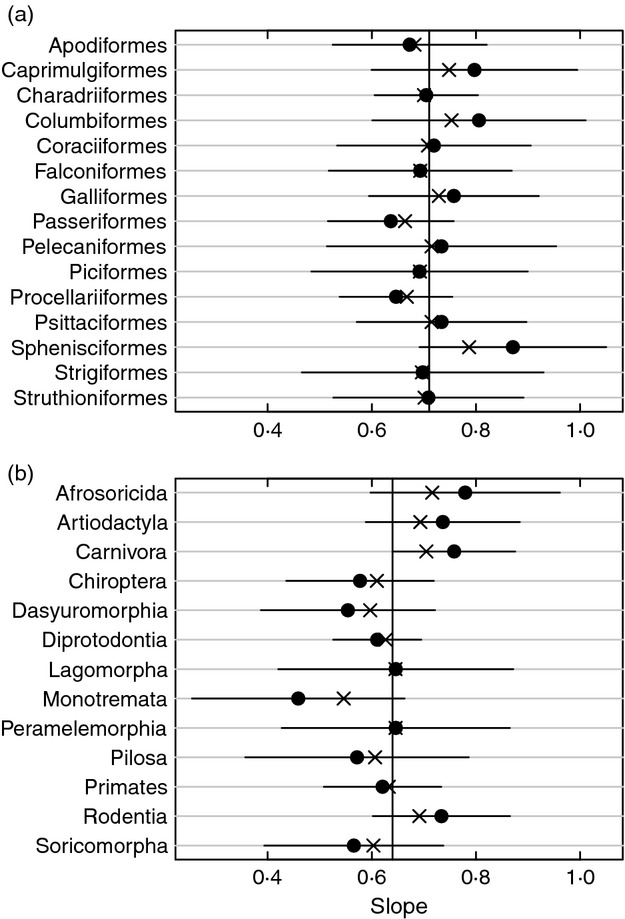
Estimates of slope by order for (a) birds and (b) mammals. Filled circles and horizontal lines mark the best model’s random-effects estimates together with their 95% confidence intervals, offset by the best model’s fixed-effects estimates. Vertical lines mark the model-averaged fixed-effects estimate. Crosses mark model-averaged values per order, computed by summing model-averaged fixed-effect slopes and model-averaged conditional means of the random effect of order on slope. Models without a random effect of order on slope were treated as having a conditional mean of zero. As far as we are aware, it is not possible to compute model-averaged confidence intervals on predictions that include random effects, so the crosses are not accompanied by confidence intervals.

Theories exist that try to explain variation in the exponent, *b*. These include the theories of Savage, Deeds & Fontana (52) and Kolokotrones *et al*. (35), the metabolic-level boundaries hypothesis (Glazier 25, 27), the cell metabolism hypothesis (Kozłstrokowski, Konarzewski & Gawelczyk 36) and the quantum metabolism theory reviewed by Agutter & Tuszynski (1). Many theories fit at least some aspects of empirical data, so it is hard to resoundingly disprove any of them. Nevertheless, our heterogeneity-of-slope results do partly support some theories and partly contradict others. For example, several theories predict that *b* will take a value between 2/3 and 1 (Kozłstrokowski, Konarzewski & Gawelczyk 36; Glazier 25; van der Meer 39). These theories are not entirely consistent with our data, since, taking order-level slopes to be normally distributed with standard deviation 0·0871 and mean 0·71 for birds and 0·64 for mammals, as estimated by our model, and then using quantiles, we find that 31% of bird orders and 62% of mammal orders are predicted to have slopes less than 2/3. The quantum metabolism theory predicts that 1/2<*b*<1. Only 5% of mammal orders and 1% of bird orders are expected by our results to have slope <1/2, so 1/2 may be a sensible choice if a lower bound is needed. Figure 2 shows order-level slope estimates provided by our best-fitting model, as well as model-average order-level slopes. The fact that few orders have confidence intervals in Fig. 2 that fall entirely below 2/3 should not be interpreted as contradicting our assessment that 31% of bird orders and 62% of mammal orders are predicted to have slopes <2/3. While the statistical methods of this study are not designed to provide great confidence about the scaling exponent for any particular order, they do strongly support the presence of substantial variation among order-level scaling exponents, both among orders for which data were included and, by inference, orders yet to be sampled. So we can say with great confidence that a substantial fraction of orders have scaling exponents below 2/3, even though we can only confidently identify a few specific orders with slope below that value.

The theories of Savage, Deeds & Fontana (52) and Kolokotrones *et al*. (35) predict that orders with smaller average body size will have shallower slope (i.e. smaller scaling exponent). We assessed this by fitting four linear regression models. Response variables were order-level slopes for birds or mammals (Fig. 2), and predictor variables were one of two measures of average order body size (making four possible combinations for four models). Average order body sizes were either the mean of the 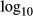-transformed body masses of individuals in our data set in the order, or the mean of the 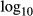-transformed body masses of the species in our data set in the order, where species log body mass was the mean of the logs of the individuals in the species. In no case was a regression trend visible; all *P*-values were >0·05. Therefore, our data provide no support for the idea that orders with smaller body size have shallower slope.

The metabolic levels boundary hypothesis (Glazier 25, 26, 27) predicts that orders of higher ‘metabolic level’ should also have shallower log FMR vs. log body mass slope. The technique used by Isaac & Carbone (31) to test the hypothesis is unfortunately flawed, because their estimates of metabolic level are not independent of body size. The output of our statistical model can be used to test the metabolic levels boundary hypothesis because it provides a measure of metabolic level that is not confounded by body size, as follows. Order-level slopes and average order body sizes were computed as in the prior paragraph, using both methods reported there for computing average order body sizes. Order-level intercepts were computed analogously to order-level slopes (Fig. 2), using model averaging. Order-level slopes and intercepts together allow the identification of an order-level regression line for log FMR vs. log body size. The metabolic level for an order was defined as the height of this line at the average order body mass minus the height of the class-level regression line at the same body mass; the class-level regression line was determined by the model-averaged fixed-effects slope and intercept for the class to which an order belongs (Aves or Mammalia). Testing for a negative correlation between order metabolic level and order slope gave significant results for birds (Pearson *R* = −0·591 or −0·584, *P* = 0·010 or 0·011, respectively, for a one-sided test using the two ways of measuring average order body size) and a nonsignificant but still negative correlation for mammals (Pearson *R* = −0·079 or −0·071, *P* = 0·399 or 0·409). Thus, our results provide some support for the metabolic levels boundary hypothesis. Poorly represented orders are expected to be affected by statistical ‘shrinkage’ (Isaac & Carbone 31), which may have reduced the strength of the effect seen here. In all cases, correlation coefficients were stronger and *P*-values lower when orders were excluded that had fewer than 10 individuals in our data set. The metabolic levels boundary hypothesis predicts clearly that there should be a negative relationship between metabolic level and slope, *b*, for data on resting or basal metabolic rates, but it also predicts a positive relationship for data measured during intensive exercise, and for the intermediate case of FMR, Glazier (27) says ‘... a negative correlation between *b* and *L* [metabolic level] should also be seen in field animals and those engaged in minimal (routine) activities, as long as maintenance costs remain a large proportion of the energy budget.’ So we add the caveat that our results support the theory if FMR can be seen as routine activity as suggested by Glazier (27), maintenance costs can be considered a large proportion of the energy budget in the field, and hence, the theory is interpreted to predict a negative correlation between *b* and *L* for FMR.

Heat dissipation limit theory does not make explicit statements about taxonomic variation in *b*, but the derivation of the theory in Speakman & Król (55) suggests ways it might be amplified to explain variation; an expanded theory could be tested against our results. The theory assumes that heat dissipation, and therefore metabolic rate, is proportional to 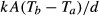, where *d* is the depth of an insulating layer (feathers, blubber or fur), *k* is the thermal conductivity of that layer, *A* is the surface area of the organism and 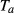 and 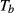 are the ambient and core body temperatures, respectively. Using empirical data and theory to write each of these components as a power function of animal mass, Speakman & Król (55) conclude that metabolic rate should be proportional to 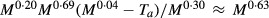. However, the component allometries, 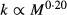, 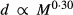, 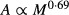, 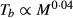, are probably subject to taxonomic heterogeneity in exponents, which would ramify through the formula to produce taxonomic heterogeneity in the scaling of metabolic rate. Assembling the appropriate data on insulating-layer depths, thermal conductivities, etc., would allow future workers to test the theory. Presumably, supply-network theories could be tested in an analogous way by examining aspects of the circulatory systems of different orders of mammals or birds, but these measurements seem harder to get than the measurements needed to test the theory of Speakman & Król (55).

Another likely rewarding avenue for future research is carrying out an analysis similar to ours but for BMR or RMR, and making comparisons with theory and between FMR and RMR results. Recent years have seen an increasing interest in the ecological and evolutionary causes and consequences of intraspecific variation in RMR (e.g. Clarke & Johnston 13; Glazier 25; Burton *et al*. 9; White, Schimpf & Seymour in press). Much data on individual resting rates are scattered in the literature, or have been partially collected, but to our knowledge, no comprehensive collection of individual measurements of RMR and body size for birds and mammals has been assembled. Most published BMR data sets contain species-averaged data. For instance, Isaac & Carbone (31) carried out an analysis like ours on a large collection of species-averaged data. White, Phillips & Seymour (60) present some individual data, but their values appear to be averages for mammal and bird species. Ehnes, Rall & Brose (19), Riveros & Enquist (51) and much work of Glazier (25) have examined individual-level data sets, but some of those works focus on clades other than birds and mammals, and the collections examined for birds and mammals are not comprehensive. White, Schimpf & Seymour (In press) studied a collection of individual measurements, but it was not intended to be a comprehensive collection, as they had different research goals. Clarke & Johnston (13) provide a large data set of individual-level measurement for fish. Burton *et al*. (9) review intraspecific variation in resting rates, including information pertinent to birds and mammals, but do not provide or analyse a comprehensive database.

Comparisons between BMR and RMR scaling and the scaling of other types of metabolic rate, including FMR, have been made by many authors, including Nagy (42), White & Seymour (63) and others. Glazier has examined the topic in depth, and his metabolic levels boundary hypothesis offers explanations of differences (Glazier 27). But compiling a comprehensive database and comparing RMR and FMR data using unified statistical models, such as ours, that simultaneously take into account central tendency scaling exponents, taxonomic variation in exponents and evolutionary nonindependence of data can probably improve understanding of the differences between RMR and FMR scaling and help develop theoretical explanations such as the metabolic levels boundary hypothesis. Scaling exponents of metabolic rate are predicted by the metabolic levels boundary hypothesis to be influenced both by volume-related constraints on energy use and production, which scale with exponent 1, and by surface-area-related constraints on fluxes of resources and waste products, which scale with exponent 2/3. At very low metabolic levels (e.g. rates measured during dormancy), surface-area constraints are not predicted to be important, so the metabolic levels boundary hypothesis predicts rates at that level will scale with exponent 1. The same scaling is predicted at very high metabolic levels (maximal metabolic rate, measured during strenuous exercise), because surface-area constraints are temporarily avoided through physiological mechanisms such as stored energy in muscle tissues and temporary tolerance to waste build-ups. At some intermediate metabolic level, surface-area constraints dominate. Therefore, the metabolic levels boundary hypothesis predicts that as metabolic level increases from minimal, through resting rates and field rates, to maximal, scaling exponents will decline from 1 to 2/3 and then will climb back to 1 again. There appears to be some variation and uncertainty in the precise level at which the minimum of 2/3 is achieved, and Glazier (27) identifies the question of how metabolic level precisely affects scaling exponents as one of several main area the metabolic levels boundary hypothesis could be developed in future work (Glazier 27, his point three on p. 125). A comprehensive and unified analysis of both RMR and BMR (and possibly other levels if sufficient data can be compiled) using appropriate statistical methods seems likely to help illuminate this and other aspects of our understanding of the true variety of metabolic scaling relationships and the reasons for this variety.

## Supporting Information

**Appendix S1.** Assembling the database. **Appendix S2.** Main models. **Appendix S3.** Within-group-centred models. **Appendix S4.** Opportunities for future improvements in the data. **Fig. S1.** Residuals against fitted values for the global model fitted by restricted maximum likelihood. **Fig. S2.** Actual values against fitted values for the global model fitted by restricted maximum likelihood. **Fig. S3.** Quantile–quantile plot of avian orders for the global model fitted by restricted maximum likelihood. **Fig. S4.** Quantile–quantile plot of mammalian orders for the global model fitted by restricted maximum likelihood. **Fig. S5.** Predictions for avian orders of the global model fitted by restricted maximum likelihood. **Fig. S6.** Predictions for mammalian orders of the global model fitted by restricted maximum likelihood. **Fig. S7.** Aquatic and nonaquatic animals. **Fig. S8.** Number of individuals measured per species. **Fig. S9.** Distributions of body masses. **Fig. S10.** Sampling locations. **Table S1.** Comparison of the main, within-group-centred and simple linear regression models. **Table S2.** Comparison of model-averaged estimates calculated using AIC and AICc. **Table S3.** Number of avian species by order. **Table S4.** Number of mammalian species by order.

Appendix S5 Database of individual FMR and body mass measurements.

Appendix S6 Database references.

## References

[b1] Agutter P, Tuszynski J (2011). Analytic theories of allometric scaling. The Journal of Experimental Biology.

[b2] Anderson KJ, Jetz W (2005). The broad-scale ecology of energy expenditure of endotherms. Ecology Letters.

[b3] Banavar J, Damuth J, Maritan A, Rinaldo A (2002). Supply-demand balance and metabolic scaling. Proceedings of the National Academy of Sciences of the United States of America.

[b4] Bates D, Mächler M, Bolker B (2011).

[b5] Bolker B, Brooks M, Clark C, Geange S, Poulsen J, Stevens M, White J (2009). Generalized linear mixed models: a practical guide for ecology and evolution. Trends in Ecology & Evolution.

[b6] Brose U, Williams R, Martinez N (2006). Allometric scaling enhances stability in complex food webs. Ecology Letters.

[b7] Brown JH, Gillooly JF, Allen AP, Savage VM, West GB (2004). Toward a metabolic theory of ecology. Ecology.

[b8] Burnham KP, Anderson DR (2002). Model Selection and Multimodel Inference: A Practical Information-Theoretic Approach.

[b9] Burton T, Killen SS, Armstrong JD, Metcalfe NB (2011). What causes the intraspecific variation in resting metabolic rate and what are its ecological consequences. Proceedings of the Royal Society. B.

[b10] Butler P, Green J, Boyd I, Speakman J (2004). Measuring metabolic rate in the field: the pros and cons of the doubly labelled water and heart rate methods. Functional Ecology.

[b11] Capellini I, Venditti C, Barton RA (2010). Phylogeny and metabolic scaling in mammals. Ecology.

[b12] Claeskens G, Hjort N (2008). Model Selection and Model Averaging.

[b13] Clarke A, Johnston NM (1999). Scaling of metabolic rate with body mass and temperature in teleost fish. Journal of Animal Ecology.

[b14] Clarke A, Rothery P, Isaac NJB (2010). Scaling of basal metabolic rate with body mass and temperature in mammals. Journal of Animal Ecology.

[b15] Crawley MJ (2007). The R Book.

[b16] Cyr H, Pace M (1993). Allometric theory: extrapolations from individuals to communities. Ecology.

[b17] Darveau C, Suarez R, Andrews R, Hochachka P (2002). Allometric cascade as a unifying principle of body mass effects on metabolism. Nature.

[b18] Dickinson EC (2003). The Howard and Moore Complete Checklist of the Birds of the World.

[b19] Ehnes R, Rall B, Brose U (2011). Phylogenetic grouping, curvature and metabolic scaling in terrestrial invertebrates. Ecology Letters.

[b20] Ernest S, Enquist B, Brown J, Charnov E, Gillooly J, Savage V, White E, Smith F, Hadly E, Haskell J, Lyons SK, Maurer BA, Niklas KJ, Tiffney B (2003). Thermodynamic and metabolic effects on the scaling of production and population energy use. Ecology Letters.

[b21] Farrell-Gray C, Gotelli N (2005). Allometric exponents support a 3/4-power scaling law. Ecology.

[b22] Feldman H, McMahon T (1983). The 3/4 mass exponent for energy metabolism is not a statistical artifact. Respiration Physiology.

[b23] Gillooly J, Allen A (2007). Changes in body temperature influence the scaling of 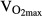 and aerobic scope in mammals. Biology Letters.

[b24] Ginzburg L, Damuth J (2008). The space-lifetime hypothesis: viewing organisms in four dimensions, literally. The American Naturalist.

[b25] Glazier D (2005). Beyond the ‘3/4-power law’: variation in the intra-and interspecific scaling of metabolic rate in animals. Biological Reviews.

[b26] Glazier D (2008). Effects of metabolic level on the body size scaling of metabolic rate in birds and mammals. Proceedings of the Royal Society. B, Biological Sciences.

[b27] Glazier D (2010). A unifying explanation for diverse metabolic scaling in animals and plants. Biological Reviews.

[b28] Greven S, Kneib T (2009). On the behaviour of marginal and conditional Akaike Information Criteria in linear mixed models. Johns Hopkins University, Dept. of Biostatistics Working Papers, 202.

[b29] Hayssen V, Lacy R (1985). Basal metabolic rates in mammals: taxonomic differences in the allometry of BMR and body mass. Comparative Biochemistry and Physiology. Part A, Molecular & Integrative Physiology.

[b30] Heusner A (1982). Energy metabolism and body size I. Is the 0.75 mass exponent of Kleiber’s equation a statistical artifact?. Respiration Physiology.

[b31] Isaac NJB, Carbone C (2010). Why are metabolic scaling exponents so controversial? Quantifying variance and testing hypotheses. Ecology Letters.

[b32] Kerkhoff A, Enquist B (2009). Multiplicative by nature: why logarithmic transformation is necessary in allometry. Journal of Theoretical Biology.

[b33] Killen S, Costa I, Brown J, Gamperl A (2007). Little left in the tank: metabolic scaling in marine teleosts and its implications for aerobic scope. Proceedings of the Royal Society. B, Biological Sciences.

[b34] Kleiber M (1932). Body size and metabolism. Hilgardia.

[b35] Kolokotrones T, Savage V, Deeds E, Fontana W (2010). Curvature in metabolic scaling. Nature.

[b36] Kozłstrokowski J, Konarzewski M, Gawelczyk A (2003). Cell size as a link between noncoding DNA and metabolic rate scaling. Proceedings of the National Academy of Sciences of the United States of America.

[b37] McNab B (2008). An analysis of the factors that influence the level and scaling of mammalian BMR. Comparative Biochemistry and Physiology. Part A, Molecular & Integrative Physiology.

[b38] McNab B (2009). Ecological factors affect the level and scaling of avian BMR. Comparative Biochemistry and Physiology. Part A, Molecular & Integrative Physiology.

[b39] van der Meer J (2006). Metabolic theories in ecology. Trends in Ecology & Evolution.

[b40] Moran D, Wells R (2007). Ontogenetic scaling of fish metabolism in the mouse-to-elephant mass magnitude range. Comparative Biochemistry and Physiology. Part A, Molecular & Integrative Physiology.

[b41] Müller D, Codron D, Werner J, Fritz J, Hummel J, Griebeler E, Clauss M (2012). Dichotomy of eutherian reproduction and metabolism. Oikos.

[b42] Nagy KA (2005). Field metabolic rate and body size. The Journal of Experimental Biology.

[b43] Nagy KA, Girard IA, Brown TK (1999). Energetics of free-ranging mammals, reptiles and birds. Annual Review of Nutrition.

[b44] Peters RH (1983). The Ecological Implications of Body Size.

[b45] Pinheiro JC, Bates DM (2000). Mixed-effects Models in S and S-PLUS.

[b46] van de Pol M, Wright J (2009). A simple method for distinguishing within–versus between–subject effects using mixed models. Animal Behaviour.

[b47] R Development Core Team (2011). R: A Language and Environment for Statistical Computing.

[b48] Reuman D, Mulder C, Banasěk-Richter C, Cattin Blandenier M, Breure A, Hollander H, Kneitel J, Raffaelli D, Woodward G, Cohen J (2009). Allometry of body size and abundance in 166 food webs. Advances in Ecological Research.

[b49] Reuman D, Mulder C, Raffaelli D, Cohen J (2008). Three allometric relations of population density to body mass: theoretical integration and empirical tests in 149 food webs. Ecology Letters.

[b50] Riek A (2008). Relationship between field metabolic rate and body weight in mammals: effect of the study. Journal of Zoology.

[b51] Riveros A, Enquist B (2011). Metabolic scaling in insects supports the predictions of the WBE model. Journal of Insect Physiology.

[b52] Savage V, Deeds E, Fontana W (2008). Sizing up allometric scaling theory. PLoS Computational Biology.

[b53] Savage V, Gillooly J, Brown J, West G, Charnov E (2004a). Effects of body size and temperature on population growth. The American Naturalist.

[b54] Savage V, Gillooly J, Woodruff W, West G, Allen A, Enquist B, Brown J (2004b). The predominance of quarter-power scaling in biology. Functional Ecology.

[b55] Speakman J, Król E (2010). Maximal heat dissipation capacity and hyperthermia risk: neglected key factors in the ecology of endotherms. Journal of Animal Ecology.

[b56] Streicher J, Cox C, Birchard G (2012). Non-linear scaling of oxygen consumption and heart rate in a very large cockroach species (*Gromphadorhina portentosa*): correlated changes with body size and temperature. The Journal of Experimental Biology.

[b57] Warton D, Wright I, Falster D, Westoby M (2006). Bivariate line-fitting methods for allometry. Biological Reviews.

[b58] West G, Brown J, Enquist B (1997). A general model for the origin of allometric scaling laws in biology. Science.

[b59] White C (2011). Allometric estimation of metabolic rates in animals. Comparative Biochemistry and Physiology. Part A, Molecular & Integrative Physiology.

[b60] White C, Phillips N, Seymour R (2006). The scaling and temperature dependence of vertebrate metabolism. Biology Letters.

[b61] White C, Schimpf NG, Cassey P The repeatability of metabolic rate declines with time. Journal of Experimental Biology.

[b62] White C, Seymour R (2003). Mammalian basal metabolic rate is proportional to body mass 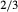. Proceedings of the National Academy of Sciences of the United States of America.

[b63] White C, Seymour R (2005). Allometric scaling of mammalian metabolism. The Journal of Experimental Biology.

[b64] White C, Terblanche J, Kabat A, Blackburn T, Chown S, Butler P (2008). Allometric scaling of maximum metabolic rate: the influence of temperature. Functional Ecology.

[b65] Wilson DE, Reeder DM (2005). Mammal Species of the World. A Taxonomic and Geographic Reference.

