# Supplementary material for: The relationship between body mass and field metabolic rate among individual birds and mammals
Source: J Anim Ecol. 2013 May 23;82(5):1009–20. doi: 10.1111/1365-2656.12086 (PMC3840704; doi:10.1111/1365-2656.12086)
Supplement: Supplementary file 1 — Appendix S1. Assembling the database. Appendix S2. Main models. Appendix S3. Within-group-centred models. Appendix S4. Opportunities for future improvements in the data. Fig. S1. Residuals against fitted values for the global model fitted by restricted maximum likelihood. Fig. S2. Actual values against fitted values for the global model fitted by restricted maximum likelihood. Fig. S3. Quantile–quantile plot of avian orders for the global model fitted by restricted maximum likelihood. Fig. S4. Quantile–quantile plot of mammalian orders for the global model fitted by restricted maximum likelihood. Fig. S5. Predictions for avian orders of the global model fitted by restricted maximum likelihood. Fig. S6. Predictions for mammalian orders of the global model fitted by restricted maximum likelihood. Fig. S7. Aquatic and nonaquatic animals. Fig. S8. Number of individuals measured per species. Fig. S9. Distributions of body masses. Fig. S10. Sampling locations. Table S1. Comparison of the main, within-group-centred and simple linear regression models. Table S2. Comparison of model-averaged estimates calculated using AIC and AICc. Table S3. Number of avian species by order. Table S4. Number of mammalian species by order. [file jane0082-1009-sd1.pdf]

The relationship between body mass and field metabolic rate among individual birds and mammals

Lawrence N Hudson   Nick J B Isaac   Daniel C Reuman

Appendix S1. Assembling the database

Our criteria for inclusion are:

1. body mass, M, measured;
2. field metabolic rate, FMR, measured in the field or in field-like captivity, using the doubly labelled water method;
3. M and FMR presented for individual animals.

We omitted data when body mass was estimated rather than measured (e.g. some data from Costa & Prince 1987). Data presented graphically was digitised using DigitizeIt 1.5 (Digital River 2011).

The doubly labelled water method estimates volume CO_2 exhaled, which can be converted to energy by multiplying by an energy equivalent, the value of which is diet dependent (Butler *et al.* 2004). Bryant (1997) studied *Turdus merula*, *Muscicapa striata* and *Parus major* and presented just volume CO_2 exhaled. We used an energy equivalent value of 26.8 J/cm^3 CO_2, taken from Ricklefs & Williams (1984), a study of *Sturnus vulgaris*, which has a similar diet to the three species investigated by Bryant (1997). Utter & LeFebvre (1973) used two plausible energy equivalent values for their study of *Progne subis*, resulting in two different estimates of FMR. We took the mean of these two estimates.

The same measurements of three individuals of *Calidris minuta* are presented by both Piersma *et al.* (2003) and Tulp *et al.* (2009). We included these data only once. Williams (1987) measured FMR of *Passerculus sandwichensis* in 1981 and 1982. The presentation of the data is not completely clear with regard to whether the same individuals were measured in both years. In addition to this, Williams (1987) classified individuals as either being territorial, incubating eggs or rearing young and it is possible that individuals were measured performing more than one activity in the same year.

To avoid including pseudo-replicated data, we took data only for those individuals that were rearing young in 1982. Similarly, Williams (1988) studied *Tachycineta bicolor* engaged in a range of activities in 1981 and 1982; again, we took data only for individuals rearing young in 1982.

We took taxonomy for mammals from Wilson & Reeder (2005) and for birds from Dickinson (2003). We ignored the sub-species level of taxonomy. Pontzer *et al.* (2010) studied hybrids of Bornean (*Pongo pygmaeus*) and Sumatran (*Pongo abelii*) orangutans. We treated these as *Pongo pygmaeus*. Simmen *et al.* (2010) measured individuals from a population of *Eulemur fulvus rufus* that hybridised with some introduced *Eulemur collaris*. We treated these as *Eulemur fulvus*.

Appendix S2. Main models

The 27 mixed-effects models (discussed in 'The main set of models', in the main text) had fixed effects of taxonomic class on both slope and intercept. The most complex, 'global' model had random effects of order, family and species on both slope and intercept:

$$\begin{aligned}\log_{10}(\mathrm{FMR}_{ijkl}) =& a_c + \alpha_{co} + \alpha_{cof} + \alpha_{cofb} + \alpha_t + \\ & (b_c + \beta_{co} + \beta_{cof} + \beta_{cofb} + \beta_t) \log_{10}(\mathrm{M}_{ijkl}) + \\ & \epsilon_{ijkl},\end{aligned} \tag{S1}$$

$$\begin{aligned}(\alpha_{co}, \beta_{co}) &\sim \mathcal{N}(0, \Sigma_{co}), \\ (\alpha_{cof}, \beta_{cof}) &\sim \mathcal{N}(0, \Sigma_{cof}), \\ (\alpha_{cofb}, \beta_{cofb}) &\sim \mathcal{N}(0, \Sigma_{cofb}), \\ (\alpha_t, \beta_t) &\sim \mathcal{N}(0, \Sigma_t), \\ \epsilon_{ijkl} &\sim \mathcal{N}(0, \sigma^2).\end{aligned}$$

FMR_{ijkl} and M_{ijkl} are the FMR and M of the lth individual in the kth species, j^{th} family and i^{th} order. As described in the main text, the $\log_{10}(M_{ijkl})$ have been centred by subtraction of the grand mean. c, o, f and b are taxonomic levels: class, order, family and species. t is the study from which the data point was taken. a_c and b_c are fixed effects of class on intercept and slope respectively. Fixed effects are estimated for each class in the data so the model has four fixed-effects parameters: $a_c = a_{Aves}$,

$a_c = a_{Mammalia}$, $b_c = b_{Aves}$ and $b_c = b_{Mammalia}$. α_{co}, α_{cof} and α_{cofb} are random effects on intercept of order, family and species. α_t is the random effect on intercept of the study from which the data point was taken. Likewise, β_{co}, β_{cof}, β_{cofb} and β_t are random effects on slope for the same taxonomic levels and for the study.

Random effects that act on just intercept are characterised by a normal distribution with mean of zero and a variance that is estimated during model fitting. Random effects that act on both slope and intercept, as do all in this global model, are characterised by bivariate normal distributions; the correlation between slope and intercept is also estimated. Σ_{co}, Σ_{cof}, Σ_{cofb} and Σ_t are symmetric, 2x2 covariance matrices and each contains three parameters. Therefore when fitting the global model in equation (S1), 17 parameters are estimated: a_{Aves}, $a_{Mammalia}$, b_{Aves}, $b_{Mammalia}$, Σ_{co}, Σ_{cof}, Σ_{cofb}, Σ_t, σ^2.

Appendix S3. Within-group-centred models

To test for systematic variation in slope at different taxonomic levels ('Supporting analyses' in the main text), we formulated 27 mixed-effects models that employed the within-group-centring method described by van de Pol & Wright (2009). This test required a model formulation that used several predictors. For brevity, we define

$$x_{ijkl} = \log_{10}(\mathrm{M}_{ijkl}), \tag{S2}$$

$$y_{ijkl} = \log_{10}(\mathrm{FMR}_{ijkl}), \tag{S3}$$

where i, j, k, l are order, family, species and individual. From these values, we computed the mean $\log_{10}$ masses at each taxonomic level. Each set of means was computed as an unweighted mean of the set of means at the level of the next higher taxonomic resolution. For instance, $\overline{x_{ijk}}$ was defined as the mean $\log_{10}$ mass of the kth species in the jth family, in the ith order, computed as the unweighted mean of x_{ijkl} as the index l varies and the other indices are fixed. From the species-level means, we computed $\overline{x_{ij}}$, the mean $\log_{10}$ mass of each of the families. Lastly, we computed $\overline{x_i}$, the mean masses of each of the orders, computed as the unweighted mean of the family means.

We formulated a model that uses the hierarchically computed means as predictors, allowing a

separate mean slope at each taxonomic level:

$$
\begin{aligned}
y_{ijkl} =& a_c + b_{c1}\overline{x_i} + b_{c2}(\overline{x_{ij}} - \overline{x_i}) + b_{c3}(\overline{x_{ijk}} - \overline{x_{ij}}) + b_{c4}(x_{ijkl} - \overline{x_{ijk}}) + \\
& (\alpha_{co} + \beta_{co}(\overline{x_{ij}} - \overline{x_i})) + \\
& (\alpha_{cof} + (\beta_{co} + \beta_{cof})(\overline{x_{ijk}} - \overline{x_{ij}})) + \\
& (\alpha_{cofb} + (\beta_{co} + \beta_{cof} + \beta_{cofb})(x_{ijkl} - \overline{x_{ijk}})) + \\
& (\alpha_t + \beta_t x_{ijkl}) + \epsilon_{ijkl},
\end{aligned}
\tag{S4}
$$

$$
\begin{aligned}
(\alpha_{co}, \beta_{co}) &\sim \mathcal{N}(0, \Sigma_{co}), \\
(\alpha_{cof}, \beta_{cof}) &\sim \mathcal{N}(0, \Sigma_{cof}), \\
(\alpha_{cofb}, \beta_{cofb}) &\sim \mathcal{N}(0, \Sigma_{cofb}), \\
(\alpha_t, \beta_t) &\sim \mathcal{N}(0, \Sigma_t), \\
\epsilon_{ijkl} &\sim \mathcal{N}(0, \sigma^2).
\end{aligned}
$$

This model has four fixed effects of class on slope: b_{c1}, b_{c1}, b_{c1} and b_{c4}.

We compared the global model of equation (S4) to our main global model (equation (S1) in) by fitting 27 models for each of these two equations, with random effects structures as described in the main text. As equations (S1) and (S4) have different fixed-effects structures, we fitted all these models using maximum likelihood. Results are in Table S1. Results demonstrate that the data do not display systematic variation of slope at lower taxonomic levels and that our main set of models derived from equation (S1) are appropriate for the data.

We here show that the fixed-effect structure of (S1) is a simplification of that of equation (S4). The fixed-effects structure of equation (S4) is

$$a_c + b_{c1}\overline{x_i} + b_{c2}(\overline{x_{ij}} - \overline{x_i}) + b_{c3}(\overline{x_{ijk}} - \overline{x_{ij}}) + b_{c4}(x_{ijkl} - \overline{x_{ijk}}).$$

Assuming that all fixed effects of class are equal ($b_{c1} = b_{c2} = b_{c3} = b_{c4} = b_c$) gives

$$a_c + b_c(\overline{x_i} + \overline{x_{ij}} - \overline{x_i} + \overline{x_{ijk}} - \overline{x_{ij}} + x_{ijkl} - \overline{x_{ijk}}).$$

Cancelling,

$$a_c + b_c(\cancel{\overline{x}_i} + \cancel{\overline{x_{ij}}} - \cancel{\overline{x}_i} + \cancel{\overline{x_{ijk}}} - \cancel{\overline{x_{ij}}} + x_{ijkl} - \cancel{\overline{x_{ijk}}}),$$

leaves

$$a_c + b_c x_{ijkl},$$

which is the fixed effects structure of equation (S1).

Appendix S4. Opportunities for future improvements in the data

Although our database is a comprehensive collection of published individual-level measurements for birds and mammals, it could be improved as a representation of extant species in three ways. First, species in our database are non-randomly sampled from orders and many orders are unrepresented or poorly represented. Numbers of bird and mammal species in our database are compared to numbers of extant bird and mammal species, by order, in Tables S3 and S4. An example of a poorly-represented order is Piciformes: our database contains just *Melanerpes formicivorus* (the acorn woodpecker) with a mean mass of 82.25g, but the order contains 396 species (Dickinson 2003) ranging from *Pogoniulus simplex* (the green tinkerbird), with a mean mass of 8.8g, to *Ramphastos swainsonii* (the chestnut-mandibled toucan), with a mean mass of 709g (Dunning 2008 with additions of Meiri, Raia & Phillimore 2011). Many species are represented by few individuals: of the 133 species in our database, 91 are represented by ten or fewer individuals (Fig. S8). Second, while average body masses of bird species in our database are distributed broadly similarly to the distribution of average body masses of extant bird species, our database contains fewer than expected small-bodied mammals (Fig. S9). The data set of Nagy, Girard & Brown (1999), which comprises species-averaged FMR information, has a body size distribution for mammals that is broadly similar to that for extant mammals, showing that measurements have been made but have unfortunately been disproportionately reported as species averages instead of at the individual level. Nagy (2005) examined species-level FMR from 79 species of mammals whereas our database contains data from 57. Third, there is a geographic bias in the data: most observations are from Europe, North America and Australia and many diverse regions of the world are less represented (Fig. S10). Our data set is large, representing essentially all published

individual measurements, but the above-listed shortcomings indicate how the data set can be usefully further expanded.

Rank	Set	Fixed-effect slope	Random effects			K	log($\mathcal{L}$)	AIC	w	$\sum(w)$
			Order	Family	Binomial					
1	Main	Per class	I	I	S & I	13	1018.92	-2011.83	0.5369	0.5369
2	Main	Per class	S & I	I	S & I	15	1020.14	-2010.27	0.2461	0.7829
3	Main	Per class	I	S & I	S & I	15	1019.10	-2008.20	0.0871	0.8700
4	Main	Per class	S & I	S & I	S & I	17	1020.15	-2006.31	0.0338	0.9039
5	Main	Per class	I		S & I	12	1014.97	-2005.93	0.0281	0.9320
6	Main	Per class	S & I		S & I	14	1016.62	-2005.25	0.0200	0.9519
7	WGC	Per class	I	I	S & I	19	1020.98	-2003.96	0.0105	0.9624
8	Main	Per class	S & I	I	I	13	1014.78	-2003.57	0.0086	0.9710
9	WGC	Per class	S & I	I	S & I	21	1022.59	-2003.17	0.0071	0.9781
10	Main	Per class	S & I		I	12	1013.13	-2002.26	0.0045	0.9826
11	WGC	Per class	S & I	I	I	19	1019.87	-2001.75	0.0035	0.9860
12	WGC	Per class	S & I	S & I	I	21	1021.61	-2001.22	0.0027	0.9887
13	WGC	Per class	I	S & I	S & I	21	1021.12	-2000.24	0.0016	0.9903
14	WGC	Per class	S & I	S & I	S & I	23	1023.07	-2000.14	0.0015	0.9919
15	WGC	Per class	S & I		S & I	20	1020.03	-2000.06	0.0015	0.9934
16	Main	Per class	I	I	I	11	1010.96	-1999.92	0.0014	0.9947
17	Main	Per class	S & I	S & I	I	15	1014.79	-1999.57	0.0012	0.9959
18	Main	Per class	I	S & I	I	13	1012.78	-1999.57	0.0012	0.9971
19	WGC	Per class	I		S & I	18	1017.60	-1999.21	0.0010	0.9981
20	WGC	Per class	S & I		I	18	1017.15	-1998.31	0.0006	0.9987
21	WGC	Per class	I	I	I	17	1015.59	-1997.18	0.0004	0.9990
22	Main	Per class		I	S & I	12	1010.43	-1996.87	0.0003	0.9993
23	Main	Per class	I		I	10	1008.39	-1996.77	0.0003	0.9996
24	WGC	Per class	I	S & I	I	19	1017.04	-1996.09	0.0002	0.9998
25	WGC	Per class		I	S & I	18	1014.90	-1993.80	<0.0001	0.9999
26	WGC	Per class	I		I	16	1012.68	-1993.35	<0.0001	0.9999
27	Main	Per class		S & I	S & I	14	1010.66	-1993.33	<0.0001	1.0000
28	WGC	Per class		S & I	S & I	20	1014.90	-1989.81	<0.0001	1.0000
29	Main	Per class		S & I	I	12	1004.43	-1984.87	<0.0001	1.0000
30	WGC	Per class		I	I	16	1007.89	-1983.78	<0.0001	1.0000
31	WGC	Per class		S & I	I	18	1009.71	-1983.42	<0.0001	1.0000
32	Main	Per class		I	I	10	1001.00	-1982.00	<0.0001	1.0000
33	Main	Per class	S & I	I		12	995.76	-1967.52	<0.0001	1.0000
34	Main	Per class	S & I	S & I		14	996.53	-1965.05	<0.0001	1.0000
35	WGC	Per class	S & I	I		18	1000.39	-1964.79	<0.0001	1.0000
36	WGC	Per class	S & I	S & I		20	1002.39	-1964.78	<0.0001	1.0000
37	Main	Per class	I	S & I		12	994.30	-1964.59	<0.0001	1.0000
38	Main	Per class	I	I		10	992.03	-1964.07	<0.0001	1.0000
39	Main	Per class	S & I			11	991.09	-1960.18	<0.0001	1.0000
40	WGC	Per class	I	S & I		18	997.85	-1959.71	<0.0001	1.0000
41	WGC	Per class	I	I		16	995.51	-1959.02	<0.0001	1.0000
42	WGC	Per class			S & I	17	996.40	-1958.80	<0.0001	1.0000
43	Main	Per class			S & I	11	990.36	-1958.73	<0.0001	1.0000
44	WGC	Per class	S & I			17	995.46	-1956.92	<0.0001	1.0000
45	Main	Per class	I			9	984.66	-1951.32	<0.0001	1.0000

Rank	Set	Fixed-effect slope	Random effects			K	log($\mathcal{L}$)	AIC	w	$\sum(w)$
			Order	Family	Binomial					
46	Main	Per class			I	9	984.47	-1950.95	<0.0001	1.0000
47	Main	Per class		S & I		11	986.39	-1950.78	<0.0001	1.0000
48	Main	Per class		I		9	984.30	-1950.59	<0.0001	1.0000
49	WGC	Per class			I	15	990.25	-1950.50	<0.0001	1.0000
50	WGC	Per class		S & I		17	991.45	-1948.90	<0.0001	1.0000
51	WGC	Per class		I		15	988.76	-1947.51	<0.0001	1.0000
52	WGC	Per class	I			15	988.26	-1946.51	<0.0001	1.0000
53	WGC	Per class				14	956.28	-1884.56	<0.0001	1.0000
54	Main	Per class				8	939.57	-1863.13	<0.0001	1.0000
55	LR	Per class				5	146.33	-282.66	<0.0001	1.0000
56	LR	Single				4	135.51	-263.01	<0.0001	1.0000
57	LR	2/3				3	128.38	-250.76	<0.0001	1.0000
58	LR	3/4				3	71.01	-136.03	<0.0001	1.0000

Table S1: Comparison of the main (Main), within-group centred (WGC) and simple linear regression (LR) models. All models were fitted using maximum likelihood for this comparison. The mixed-effects models had random effects of taxonomy on either intercept (I) or slope and intercept (S & I). K is the number of parameters and $\mathcal{L}$ is maximum likelihood.

Method	Fixed-effects slopes (95% conf. int.)	
	Aves	Mammalia
AIC	0.710 (0.625,0.795)	0.640 (0.564,0.716)
AIC_c	0.710 (0.625,0.794)	0.640 (0.564,0.715)

Table S2: Comparison of model-averaged estimates calculated using AIC and AIC_c. The main models were fitted using restricted maximum likelihood for this analysis.

Order	Extant species	Species in our data	% extant species in our data
Anseriformes	157	0	0.00
Apodiformes	425	5	1.18
Apterygiformes	3	0	0.00
Bucerotiformes	51	0	0.00
Caprimulgiformes	115	1	0.87
Casuariiformes	4	0	0.00
Charadriiformes	344	17	4.94
Ciconiiformes	124	0	0.00
Coliiformes	6	0	0.00
Columbiformes	298	1	0.34
Coraciiformes	148	1	0.68
Cuculiformes	138	0	0.00
Falconiformes	296	2	0.68
Galliformes	287	3	1.05
Gaviiformes	5	0	0.00
Gruiformes	183	0	0.00
Musophagiformes	23	0	0.00
Passeriformes	5705	27	0.47
Pelecaniformes	62	2	3.23
Phoenicopteriformes	5	0	0.00
Piciformes	396	1	0.25
Podicipediformes	19	0	0.00
Procellariiformes	107	6	5.61
Psittaciformes	352	4	1.14
Pteroclidiformes	16	0	0.00
Rheiformes	2	0	0.00
Sphenisciformes	17	4	23.53
Strigiformes	194	1	0.52
Struthioniformes	1	1	100.00
Tinamiformes	46	0	0.00
Trogoniformes	39	0	0.00
Turniciformes	16	0	0.00
Upupiformes	9	0	0.00
All 33 orders	9593	76	0.79

Table S3: Number of avian species by order. Counts in ‘Extant species’ taken from Dickinson (2003).

Order	Extant species	Species in our data	% extant species in our data
Afrosoricida	51	1	1.96
Artiodactyla	239	5	2.09
Carnivora	286	10	3.50
Cetacea	84	0	0.00
Chiroptera	1116	7	0.63
Cingulata	21	0	0.00
Dasyuromorphia	71	2	2.82
Dermoptera	2	0	0.00
Didelphimorphia	87	0	0.00
Diprotodontia	143	13	9.09
Erinaceomorpha	24	0	0.00
Hyracoidea	4	0	0.00
Lagomorpha	92	1	1.09
Macroscelidea	15	0	0.00
Microbiotheria	1	0	0.00
Monotremata	5	1	20.00
Notoryctemorphia	2	0	0.00
Paucituberculata	6	0	0.00
Peramelemorphia	21	1	4.76
Perissodactyla	17	0	0.00
Pholidota	8	0	0.00
Pilosa	10	1	10.00
Primates	376	6	1.60
Proboscidea	3	0	0.00
Rodentia	2277	8	0.35
Scandentia	20	0	0.00
Sirenia	5	0	0.00
Soricomorpha	428	1	0.23
Tubulidentata	1	0	0.00
All 29 orders	5415	57	1.05

Table S4: Number of mammalian species by order. Counts in 'Extant species' taken from Wilson & Reeder (2005).

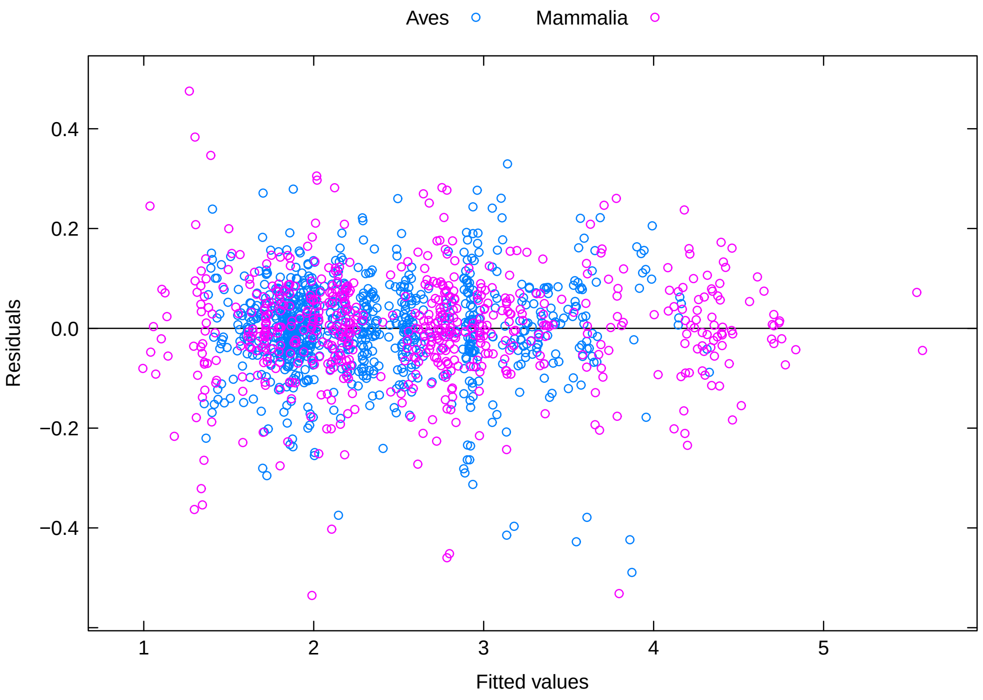

Fig. S1: Residuals against fitted values for the global model fitted by restricted maximum likelihood.

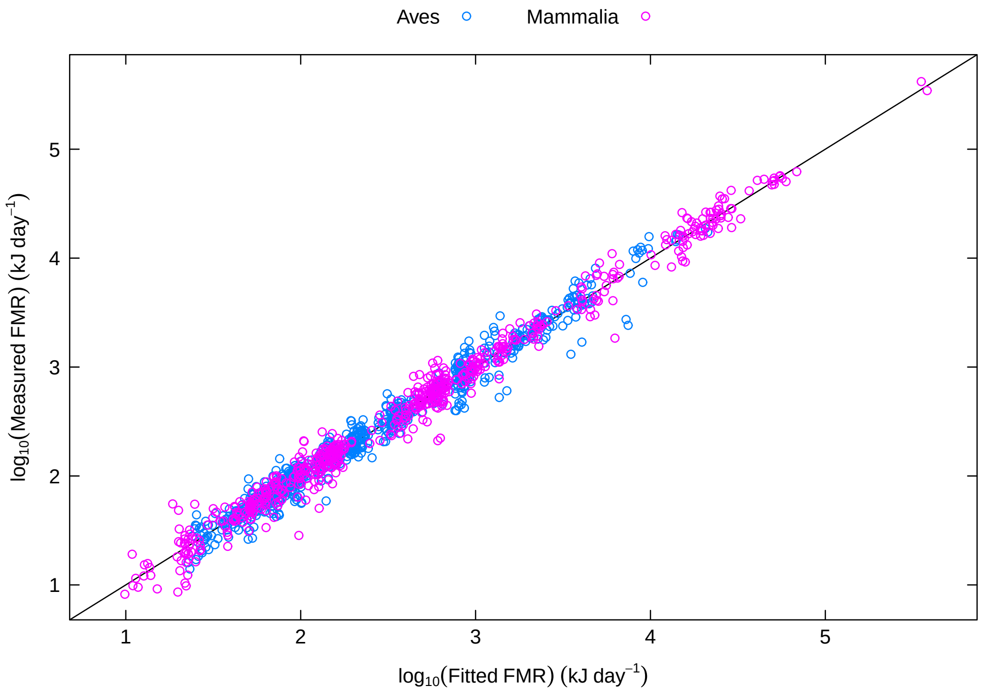

Fig. S2: Actual values against fitted values for the global model fitted by restricted maximum likelihood.

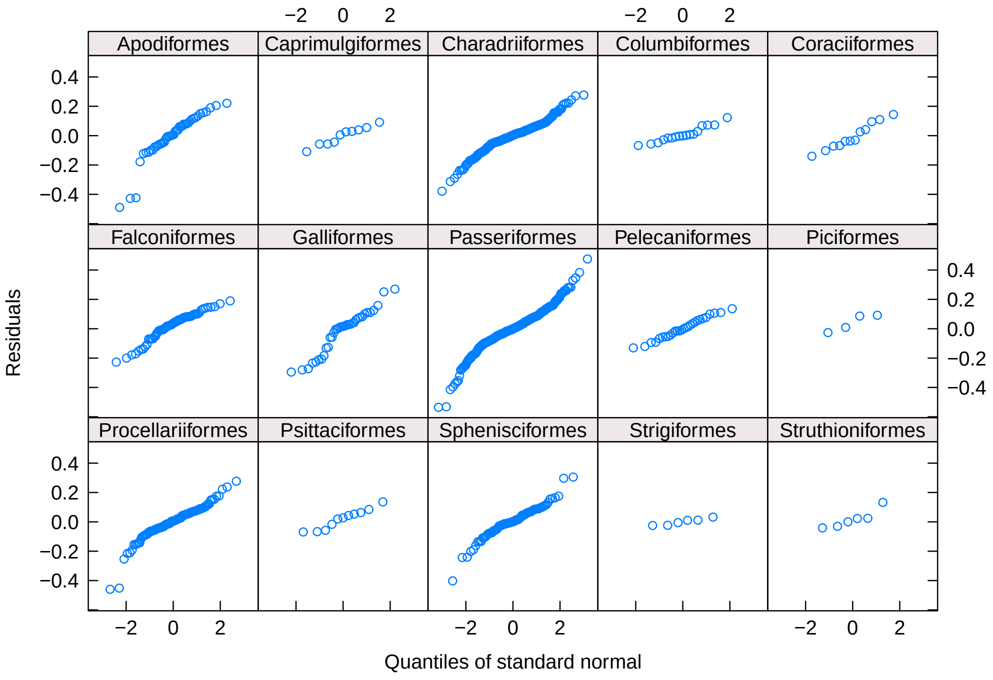

Fig. S3: Quantile-quantile plot of avian orders for the global model fitted by restricted maximum likelihood.

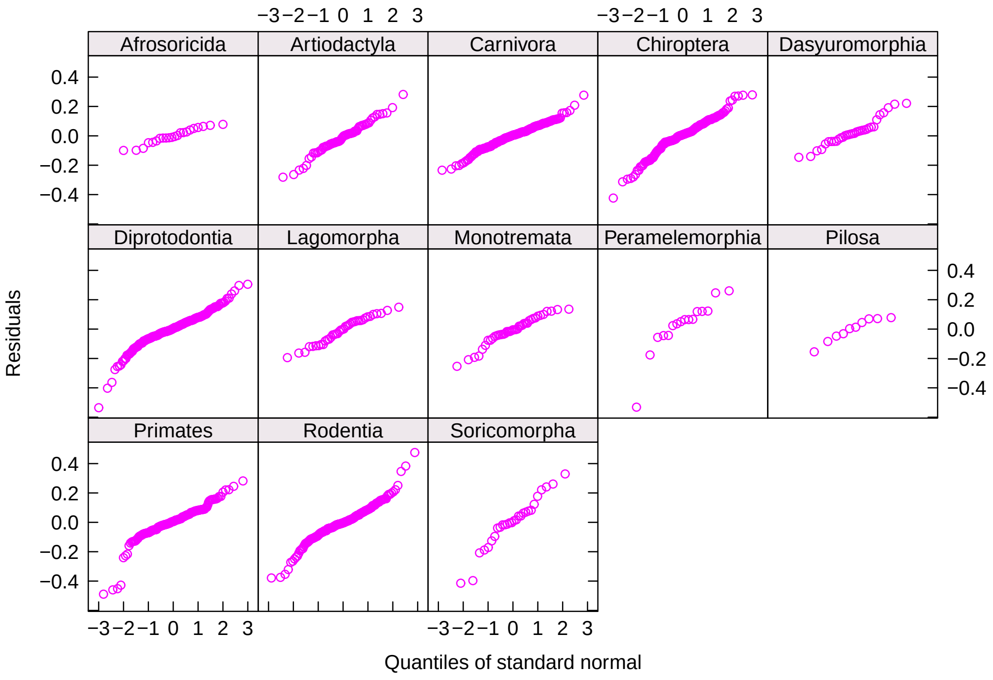

Fig. S4: Quantile-quantile plot of mammalian orders for the global model fitted by restricted maximum likelihood.

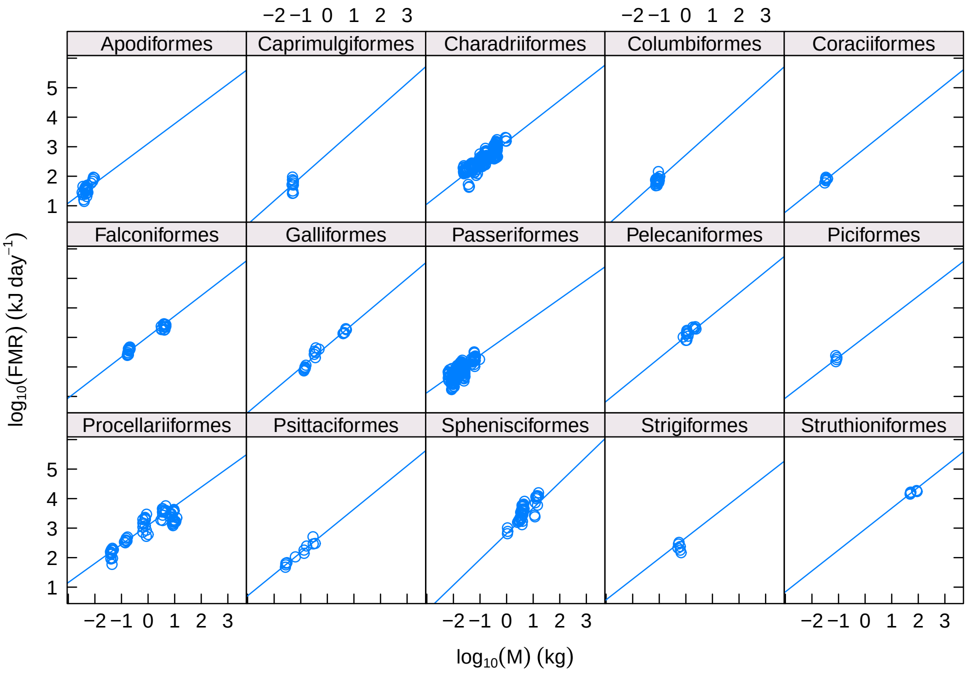

Fig. S5: Predictions for avian orders of the global model fitted by restricted maximum likelihood.

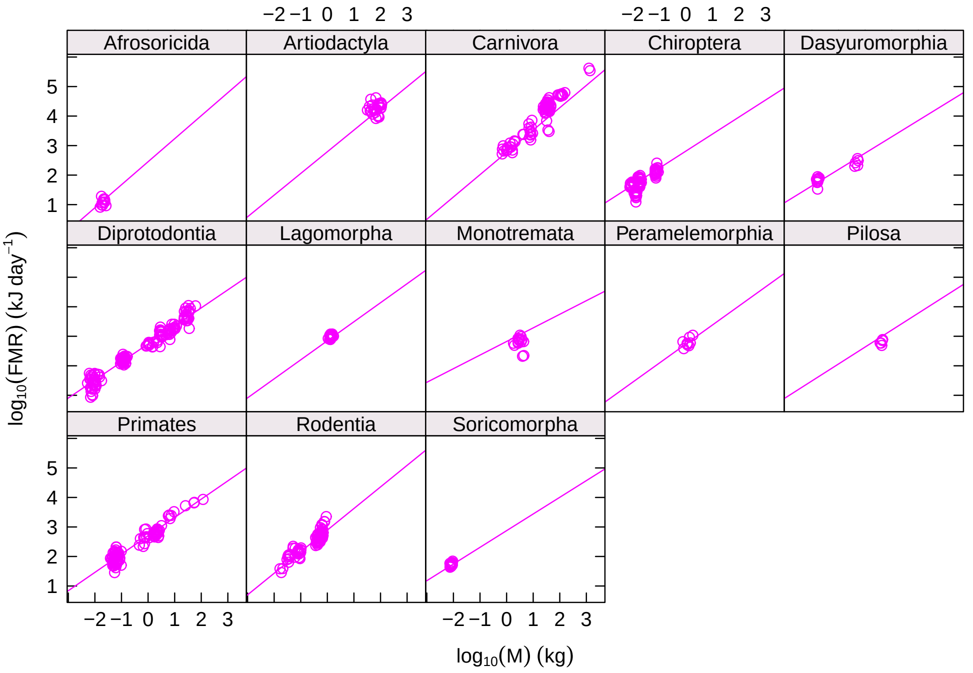

Fig. S6: Predictions for mammalian orders of the global model fitted by restricted maximum likelihood.

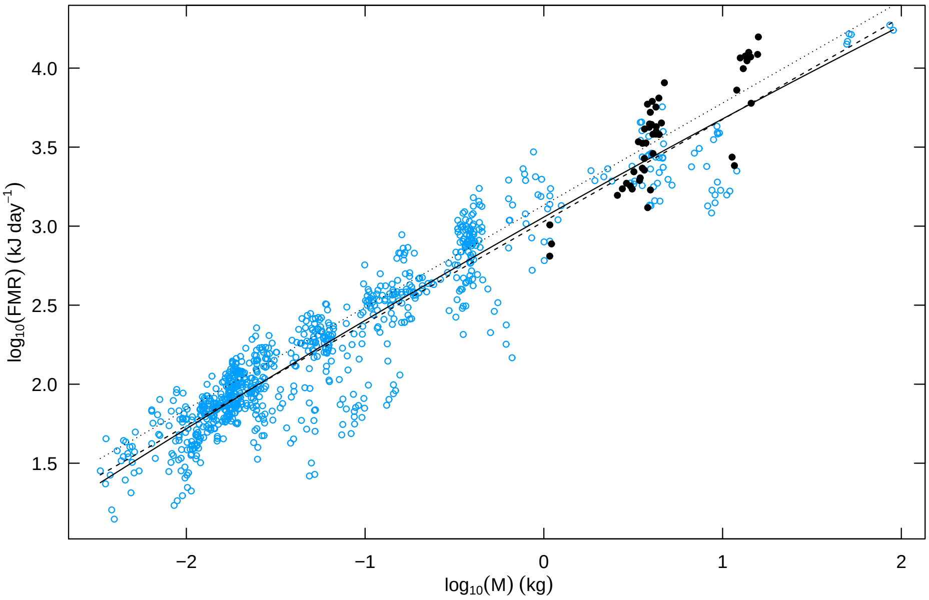

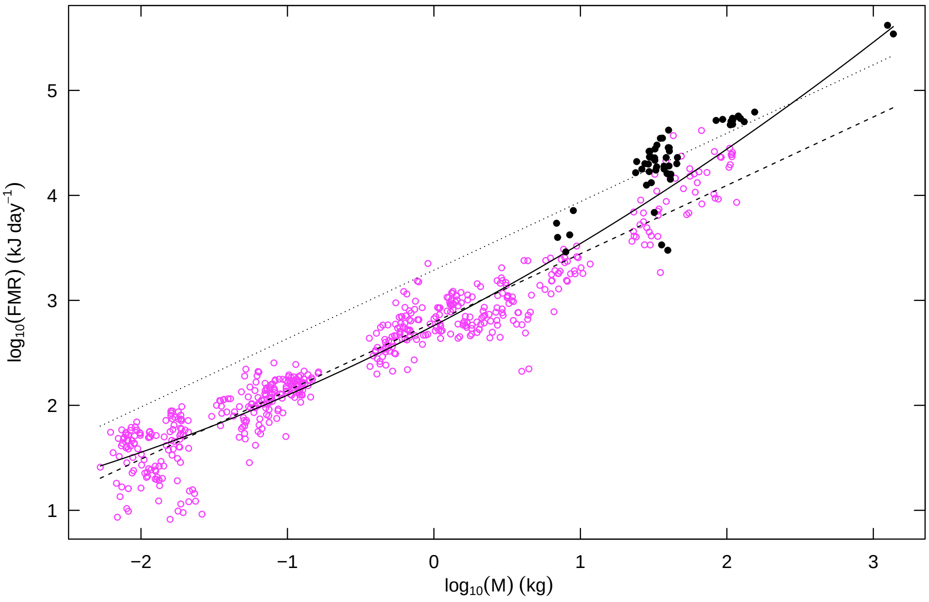

Fig. S7: Aquatic and non-aquatic birds (top) and mammals (bottom). Filled black circles show aquatic animals. The solid lines show predictions of a quadratic model, fitted to all data points. The dashed and dotted lines show predictions of a model that allows different intercepts but retains the same slope for non-aquatic and aquatic animals. Linear (single intercept) models were also fitted. For birds, AIC values were: -363.24 for the linear model with one intercept; -365.84 for the quadratic model; -371.16 for the two-intercept model. For mammals, they were: 41.57 for the linear model with one intercept; -41.27 for the quadratic model; -131.92 for the two-intercept model. For the two-intercept models, intercepts for aquatic animals were higher. There was a fairly clear-cut distinction between aquatic and non-aquatic mammals in the data set, but not so for birds. Therefore, only penguins were considered aquatic birds for the plots and analyses shown here, but we tried a variety of other ways of defining aquatic versus non-aquatic for birds, spanning a range of permissiveness in the definition of "aquatic". Results were substantially the same in all cases.

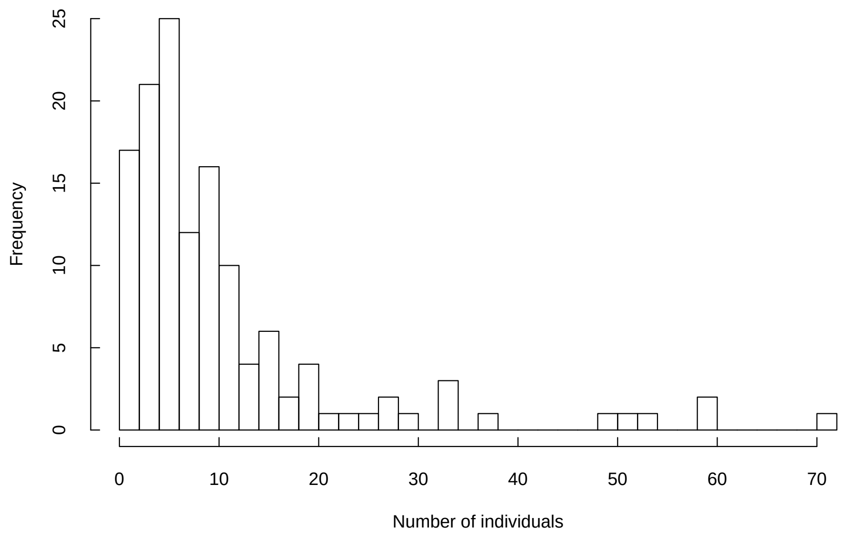

Fig. S8: Number of individuals measured per species.

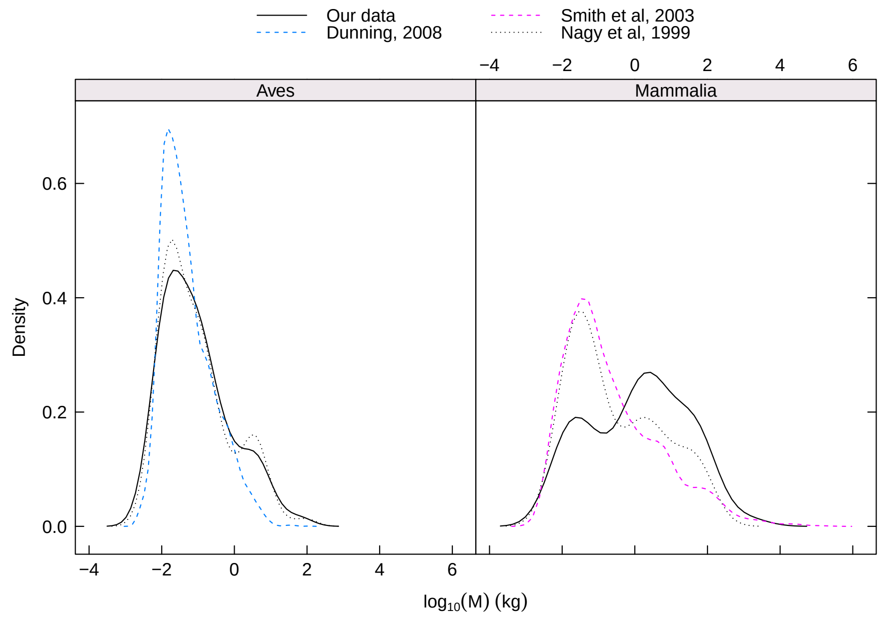

Fig. S9: Distributions of body masses. Species-averaged body masses in our data together with the avian body mass data of Dunning (2008) with additions of Meiri, Raia & Phillimore (2011) and the mammalian body mass data of Smith *et al.* (2003), the best currently available databases of body masses of birds and mammals respectively. For comparison, we include the data used by Nagy, Girard & Brown (1999).

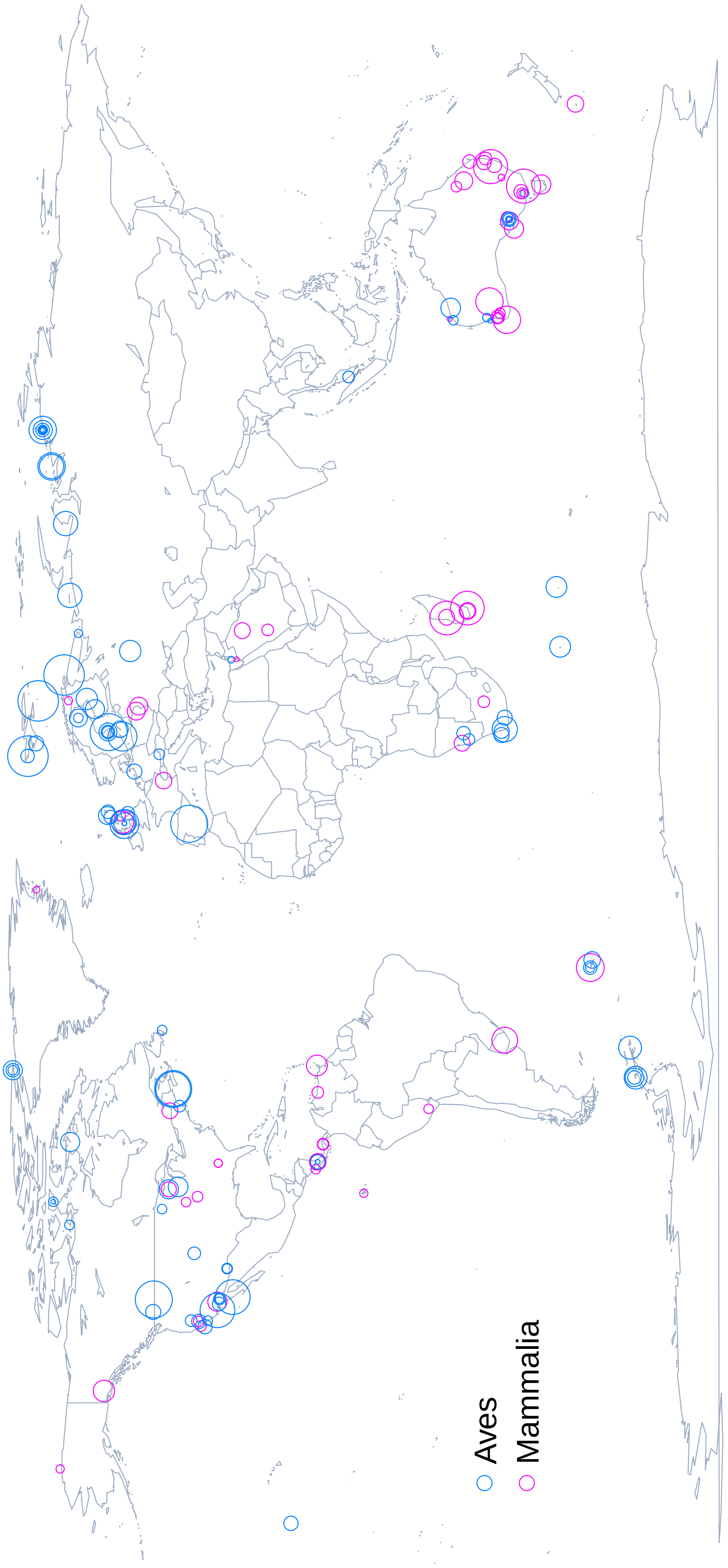

Fig. S10: Sampling locations. Each circle represents a population that was measured. The area of the circles is proportional to the number of individuals sampled. The legend shows the area for 10 individuals.

References

Bryant, D.M. (1997) Energy expenditure in wild birds, *Proceedings of the Nutrition Society*, 56, 1025–1039.

Butler, P., Green, J., Boyd, I. & Speakman, J. (2004) Measuring metabolic rate in the field: the pros and cons of the doubly labelled water and heart rate methods, *Functional Ecology*, 18, 168–183.

Costa, D.P. & Prince, P.A. (1987) Foraging energetics of gray headed albatrosses *Diomedea chrysostoma* at Bird Island, South Georgia, *Ibis*, 129, 149–158.

Dickinson, E.C., ed. (2003) *The Howard and Moore complete checklist of the birds of the world*, Christopher Helm, London, third edition.

Digital River (2011) *DigitizeIt*, Cologne, Germany.

Dunning, Jr., J.B. (2008) *CRC handbook of avian body masses*, CRC Press, 2 edition.

Meiri, S., Raia, P. & Phillimore, A. (2011) Slaying dragons: limited evidence for unusual body size evolution on islands, *Journal of Biogeography*, 38, 80–100.

Nagy, K.A. (2005) Field metabolic rate and body size, *The Journal of Experimental Biology*, 208, 1621–1625.

Nagy, K.A., Girard, I.A. & Brown, T.K. (1999) Energetics of free-ranging mammals, reptiles and birds, *Annual Review of Nutrition*, 19, 247–277.

Piersma, T., Lindström, Ä., Drent, R.H., I, T., Jukema, J., Morrison, R.I.G., Reneerkens, J., Schekkerman, H. & Visser, G.H. (2003) High daily energy expenditure of incubating shorebirds on high Arctic tundra: a circumpolar study, *Functional Ecology*, 17, 356–362.

van de Pol, M. & Wright, J. (2009) A simple method for distinguishing within–versus between–subject effects using mixed models, *Animal Behaviour*, 77, 753–758.

Pontzer, H., Raichlen, D.A., Shumaker, R.W., Ocobock, C. & Wich, S.A. (2010) Metabolic adaptation for low energy throughput in orangutans, *Proceedings of the National Academy of Sciences of the United States of America*, 107, 14048–14052.

Ricklefs, R. & Williams, J. (1984) Daily energy expenditure and water-turnover rate of adult European starlings (*Sturnus vulgaris*) during the nesting cycle, *The Auk*, 101, 707–716.

Simmen, B., Bayart, F., Rasamimanana, H., Zahariev, A., Blanc, S. & Pasquet, P. (2010) Total Energy Expenditure and Body Composition in Two Free-Living Sympatric Lemurs, *PLoS One*, 5, e9860.

Smith, F., Lyons, S., Ernest, S., Jones, K., Kaufman, D., Dayan, T., Marquet, P., Brown, J. & Haskell, J. (2003) Body mass of late Quaternary mammals, *Ecology*, 84, 3403–3403.

Tulp, I., Schekkerman, H., Bruinzeel, L.W., Jukema, J., Visser, G.H. & Piersma, T. (2009) Energetic Demands During Incubation and Chick Rearing in a Uniparental and a Biparental Shorebird Breeding in the High Arctic, *The Auk*, 129, 155–164.

Utter, J.M. & LeFebvre, Eugene, A. (1973) Daily energy expenditure of purple martins (*Progne subis*) during the breeding season: estimates using (D_2O^{18}) and time budget methods, *Ecology*, 54, 597–604.

Williams, J.B. (1987) Field metabolism and food consumption of savannah sparrows during the breeding season, *The Auk*, 104, 277–289.

Williams, J.B. (1988) Field metabolism of tree swallows during the breeding season, *The Auk*, 105, 706–714.

Wilson, D.E. & Reeder, D.M., eds. (2005) *Mammal species of the world. A taxonomic and geographic reference*, Johns Hopkins University Press, third edition.
