# Supplementary material for: The relationship between body mass and field metabolic rate among individual birds and mammals
Source: J Anim Ecol. 2013 May 23;82(5):1009–20. doi: 10.1111/1365-2656.12086 (PMC3840704; doi:10.1111/1365-2656.12086)
Supplement: Appendix S6 — Database references. [file jane0082-1009-sd3.pdf]

The relationship between body mass and field metabolic rate among individual birds and mammals

Lawrence N Hudson Nick J B Isaac Daniel C Reuman

Appendix S6. Database references

Acquarone, M., Born, E.W. & Speakman, J.R. (2006) Field Metabolic Rates of Walrus (*Odobenus rosmarus*) Measured by the Doubly Labeled Water Method, *Aquatic Mammals*, 32, 363–369.

Adams, N.J., Brown, C.R. & Nagy, K.A. (1986) Energy expenditure of free ranging wandering albatrosses *Diomedea exulans*, *Physiological Zoology*, 59, 583–591.

Arnould, J.P.Y., Boyd, I.L. & Speakman, J.R. (1996) The relationship between foraging behaviour and energy expenditure in Antarctic fur seals, *Journal of Zoology*, 239, 769–782.

Ballance, L.T. (1995) Flight energetics of free-ranging red-footed boobies (*Sula sula*), *Physiological Zoology*, 68, 887–914.

Bell, G.P., Bartholomew, G.A. & Nagy, K.A. (1986) The roles of energetics, water economy, foraging behavior, and geothermal refugia in the distribution of the bat, *Macrotus californicus*, *Journal of Comparative Physiology B: Biochemical, Systemic, and Environmental Physiology*, 156, 441–450.

Berteaux, D., Thomas, D.W., Bergeron, J.M. & Lapierre, H. (1996) Repeatability of daily field metabolic rate in female meadow voles (*Microtus pennsylvanicus*), *Functional Ecology*, 10, 751–759.

Bradshaw, S.D. & Bradshaw, F.J. (1999) Field energetics and the estimation of pollen and nectar intake in the marsupial honey possum, *Tarsipes rostratus*, in heathland habitats of South-Western Australia, *Journal of Comparative Physiology B: Biochemical, Systemic, and Environmental Physiology*, 169, 569–580.

Bryant, D.M. (1997) Energy expenditure in wild birds, *Proceedings of the Nutrition Society*, 56, 1025–1039.

Bryant, D.M., Hails, C.J. & Tatner, P. (1984) Reproductive energetics of 2 tropical bird species, *The Auk*, 101, 25–37.

Bryce, J.M., Speakman, J.R., Johnson, P.J. & Macdonald, D.W. (2001) Competition between Eurasian red and introduced Eastern grey squirrels: the energetic significance of body-mass differences, *Proceedings of the Royal Society B - Biological Sciences*, 268, 1731–1736.

Burness, G.P., Ydenberg, R.C. & Hochachka, P.W. (2001) Physiological and biochemical correlates of brood size and energy expenditure in tree swallows, *The Journal of Experimental Biology*, 204, 1491–1501.

Cairns, D.P., Montevecchi, W.A., Birt-Friesen, V.L. & Macko, S.A. (1990) Energy expenditures, activity budgets, and prey harvest of breeding common murres, *Studies in Avian Biology*, 14, 84–92.

Carlson, A., Moreno, J. & Alatalo, R.V. (1993) Winter metabolism of coniferous forest tits Paridae under arctic conditions: a study with doubly labeled water, *Ornis Scandinavica*, 24, 161–164.

Cooper, C.E., Wither, P.C. & Bradshaw, S.D. (2003) Field metabolic rate and water turnover of the numbat (*Myrmecobius fasciatus*), *Journal of Comparative Physiology B: Biochemical, Systemic, and Environmental Physiology*, 173, 687–693.

Costa, D.P., Croxall, J.P. & Duck, C.D. (1989) Foraging energetics of Antarctic fur seals in relation to changes in prey availability, *Ecology*, 70, 596–606.

Costa, D.P., Dann, P. & Disher, W. (1986) Energy requirements of free ranging little penguin, *Eudyptula minor*, *Comparative Biochemistry and Physiology Part A: Molecular & Integrative Physiology*, 85, 135–138.

Costa, D.P. & Gales, N.J. (2000) Foraging energetics and diving behavior of lactating New Zealand sea lions, *Phocarctos hookeri*, *The Journal of Experimental Biology*, 203, 3655–3665.

Costa, D.P. & Prince, P.A. (1987) Foraging energetics of gray headed albatrosses *Diomedea chrysostoma* at Bird Island, South Georgia, *Ibis*, 129, 149–158.

Costa, D.P. & Trillmich, F. (1988) Mass changes and metabolism during the perinatal fast: a comparison between Antarctic (*Arctocephalus gazella*) and Galpagos fur seals (*Arctocephalus galapagoensis*), *Physiological Zoology*, 61, 160–169.

Coup, R.N. & Perkins, P.J. (1999) Field metabolic rate of wild turkeys in winter, *Canadian Journal of Zoology*, 77, 1075–1082.

Crocker, D.E., Kofahl, N., Fellers, G.D., Gates, N.B. & Houser, D.S. (2007) High rates of energy expenditure and water flux in free-ranging Point Reyes Mountain beavers *Aplodontia rufa phaea*, *Physiological and Biochemical Zoology*, 80, 635–642.

Culik, B.M. & Wilson, R.P. (1992) Field metabolic rates of instrumented Adélie penguins using double-labelled water, *Journal of Comparative Physiology B: Biochemical, Systemic, and Environmental Physiology*, 162, 567–573.

Degen, A.A., Pinshow, B., Yosef, R., Kam, M. & Nagy, K.A. (1992) Energetics and growth rate of northern shrike (*Lanius excubitor*) nestlings, *Ecology*, 73, 2273–2283.

Dekar, M.P., Magoulick, D.D. & Beringer, J. (2010) Bioenergetics assessment of fish and crayfish consumption by river otter (*Lontra canadensis*): integrating prey availability, diet, and field metabolic rate, *Canadian Journal of Fisheries and Aquatic Sciences*, 67, 1439–1448.

Drack, S., Ortmann, S., Bührmann, N., Schmid, J., Warren, R.D., Heldmaier, G. & Ganzhorn, J.U. (1999) Field metabolic rate and the cost of ranging of the red-tailed sportive lemur (*Lepilemur ruficaudatus*), in B. Rakotosamimanana, H. Rasaminmanana, J.U. Ganzhorn & S.M. Goodman, eds., *New directions in lemur studies*, chapter 5, Kluwer Academic / Plenum Publishers, pp. 83–91.

Dykstra, C.R. & Karasov, W.H. (1993) Daily energy expenditure by nestling house wrens, *The Condor*, 95, 1028–1030.

Dykstra, C.R., Karasov, W.H., Meyer, M.W. & Warnke, D.K. (2001) Daily energy expenditure of nestling Bald Eagles in northern Wisconsin, *The Condor*, 103, 175–179.

Ellis, W.A.H., Melzer, A., Green, B., Newgrain, K., Hindell, M.A. & Carrick, F.N. (1995) Seasonal variation in water flux, field metabolic rate and food consumption of free-ranging koalas (*Phascolarctos cinereus*), *Australian Journal of Zoology*, 43, 59–68.

Engstrand, S.M., Ward, S. & Bryant, D.M. (2002) Variable energetic responses to clutch size manipulations in white-throated dippers *Cinclus cinclus*, *Journal of Avian Biology*, 33, 371–379.

Evans, M., Green, B. & Newgrain, K. (2003) The field energetics and water fluxes of free-living wombats (Marsupialia: Vombatidae), *Oecologia*, 137, 171–180.

Fleming, T.H. (1988) Energetics, in T.H. Fleming, ed., *The short-tailed fruit bat: a study in plant-animal interactions*, chapter 8, The University of Chicago Press, Chicago, pp. 217–238.

Foley, W.J., Kehl, J.C., Nagy, K.A., Kaplan, I.R. & Borsboom, A.C. (1990) Energy and water metabolism in free-living greater gliders, *Petauroides volans*, *Australian Journal of Zoology*, 38, 1–9.

Furness, R.W. & Bryant, D.M. (1996) Effect of wind on field metabolic rates of breeding northern fulmars, *Ecology*, 77, 1181–1188.

Fyhn, M., Gabrielsen, G.W., Nordøy, E.S., Moe, B., Langseth, I. & Bech, C. (2001) Individual variation in field metabolic rate of kittiwakes (*Rissa tridactyla*) during the chick-rearing period, *Physiological and Biochemical Zoology*, 74, 343–355.

Gabrielsen, G.W., Mehlum, F. & Nagy, K.A. (1987) Daily energy expenditure and energy utilization of free-ranging black-legged kittiwakes, *The Condor*, 89, 126–132.

Gabrielsen, G.W., Taylor, J.R.E., Konarzewski & Mehlum, F. (1991) Field and laboratory metabolism and thermoregulation in dovekies (*Alle alle*), *The Auk*, 108, 71–78.

Geffen, E., Degen, A.A., Kam, M., Hefner, R. & Nagy, K.A. (1992) Daily energy expenditure and water flux of free-living Blanfords foxes (*Vulpes cana*), a small desert carnivore, *Journal of Animal Ecology*, 61, 611–617.

Geiser, F. & Coburn, D.K. (1999) Field metabolic rates and water uptake in the blossom-bat *Syconycteris australis* (Megachiroptera), *Journal of Comparative Physiology B: Biochemical, Systemic, and Environmental Physiology*, 169, 133–138.

Gilbert, J.H., Zollner, P.A., Green, A.K., Wright, J.L. & Karasov, W.H. (2009) Seasonal Field Metabolic Rates of American Martens in Wisconsin, *American Midland Naturalist*, 162, 327–334.

Goldstein, D.L. & Nagy, K.A. (1985) Resource utilization by desert quail: time and energy, food and water, *Ecology*, 66, 378–387.

Gotaas, G., Milne, E., Haggarty, P. & Tyler, N.J.C. (2000) Energy expenditure of free-living reindeer estimated by the doubly-labelled water method, *Rangifer*, 20, 211–219.

Green, B., Griffiths, M. & Newgrain, K. (1992) Seasonal patterns in water, sodium and energy turnover in free-living echidnas *Tachyglossus aculeatus*, *Journal of Zoology*, 227, 351–365.

Grenot, C., Pascal, M., Buscarlet, L., Francaz, J.M. & Sellami, M. (1984) Water and energy balance in the water vole (*Arvicola terrestris sherman*) in the laboratory and in the field (Haut-Doubs, France), *Comparative Biochemistry and Physiology Part A: Molecular & Integrative Physiology*, 78, 185–196.

Haggarty, P., Robinson, J.J., Ashton, J., Milne, E., Adam, C.L., Kyle, C.E., Christie, S.L. & Midwood, A.J. (1998) Estimation of energy expenditure in free-living red deer (*Cervus elaphus*) with the doubly-labeled water method, *British Journal of Nutrition*, 80, 263–272.

von Helversen, O. & Reyer, H.U. (1984) Nectar intake and energy expenditure in a flower visiting bat, *Oecologia*, 63, 178–184.

Hodum, P.J., Sydeman, W.J., Visser, G.H. & Weathers, W.W. (1998) Energy expenditure and food requirement of Cassin's Auklets provisioning nestlings, *The Condor*, 100, 546–550.

Jönsson, K.I., Korpimäki, I.P. & Tolonen, P. (1996) Daily energy expenditure and short-term reproductive costs in free-ranging Eurasian kestrels (*Falco tinnunculus*), *Functional Ecology*, 10, 475–482.

Keller, T.M. & Visser, G.H. (1999) Daily energy expenditure of great cormorants *Phalacrocorax carbo sinensis* wintering at Lake Chiemsee, Southern Germany, *Ardea*, 87, 61–69.

Klaassen, M., Becker, P.H. & Wagener, M. (1992) Transmitter loads do not affect the daily energy expenditure of nesting common terns, *Journal of Field Ornithology*, 63, 181–185.

Kooyman, G.L., Cherel, Y., Le Maho, Y., Croxall, J.P., Thorson, P.H., Ridoux, V. & Kooyman, C.A. (1992) Diving behavior and energetics during foraging cycles in king penguins, *Ecological Monographs*, 62, 143–163.

Künkele, J., Kraus, C. & Trillmich, F. (2005) Does the unusual life listory of the precocial cavy (*Cavia magna*) translate into an exceptional field metabolic rate?, *Physiological and Biochemical Zoology*, 78, 48–54.

Kunz, T.H., Robson, S.K. & Nagy, K.A. (1998) Economy of harem maintenance in the greater spear-nosed bat, *Journal of Mammalogy*, 79, 631–642.

Mehlum, F., Gabrielson, G.W. & Nagy, K.A. (1993) Energy expenditure by black guillemots (*Cepphus grylle*) during chick rearing, *Colonial Waterbirds*, 16, 45–52.

Moreno, J., Carlson, A. & Alatalo, R.V. (1988) Winter energetics of coniferous forest tits Paridae in the north: the implications of body size, *Functional Ecology*, 2, 163–170.

Moreno, J. (1989) Variation in daily energy expenditure in nesting northern wheatears (*Oenanthe oenanthe*), *The Auk*, 106, 18–25.

Moreno, J. & Carlson, A. (1989) Clutch size and the costs of incubation in the pied flycatcher, *Ornis Scandinavica*, 20, 123–128.

Moreno, J., Cowie, R.J., Sanz, J.J. & Williams, R.S.R. (1995) Differential response by males and females to brood manipulations in the pied flycatcher: energy expenditure and nestling diet, *Journal of Animal Ecology*, 64, 721–732.

Moreno, J., Gustafsson, L., Carlson, A. & Pärt (1991) The cost of incubation in relation to clutch-size in the collared flycatcher *Ficedula albicollis*, *Ibis*, 133, 186–193.

Moreno, J. & Sanz, J.J. (1994) The relationship between the energy-expenditure during incubation and clutch size in the pied flycatcher *Ficedula hypoleuca*, *Journal of Avian Biology*, 25, 125–130.

Morrison, R.I.G., Davidson, N.C. & Piersma, T. (1997) Daily energy expenditure and water turnover of shorebirds at Alert, Ellesmere Island, N.W.T., *Canadian Wildlife Service Progress Notes*, 211, 1–8.

Nagy, K.A., Bradley, A.J. & Morris, K.D. (1990) Field metabolic rates, water fluxes, and feeding rates of quokkas, *Setonix brachyurus*, and tammars, *Macropus eugenii*, in Western Austraila, *Australian Journal of Zoology*, 37, 553–560.

Nagy, K.A., Bradshaw, S.D. & Clay, B.T. (1991) Field metabolic rate, water flux, and food requirements of short-nosed bandicoots, *Isoodon obesulus* (Marsupialia: Peramelidae), *Australian Journal of Zoology*, 39, 299–305.

Nagy, K.A., Gavrilov, V.M., Kerimov, A.B. & Ivankina, E. (1999) Relationships between field metabolic rate and territoriality in passerines, in *Acta XXII Congressus Internationalis Ornithologici, Durban, South Africa*, pp. 390–400.

Nagy, K.A., Meienberger, C., Bradshaw, S.D. & Wooller, R.D. (1995) Field metabolic rate of a small marsupial mammal, the honey possum (*Tarsipes rostratus*), *Journal of Mammalogy*, 76, 862–866.

Nagy, K.A., Sanson, G.D. & K, J.N. (1990) Comparative field energetics of two macropod marsupials and a ruminant, *Australian Wildlife Research*, 17, 591–599.

Nagy, K.A. & Bradshaw, S.D. (2000) Scaling of energy and water fluxes in free-living arid-zone australian marsupials, *Journal of Mammalogy*, 81, 962–970.

Nagy, K.A., Lee, A.K., Martin, R.W. & Fleming, M.R. (1988) Field metabolic-rate and food requirement of a small dasyurid marsupial, *Sminthopsis crassicaudata*, *Australian Journal of Zoology*, 36, 293–299.

Nagy, K.A. & Martin, R.W. (1985) Field metabolic rate, water flux, food consumption and time budget of koalas, *Phascolarctos cinereus* (Marsupialia, Phascolarctidae) in Victoria, *Australian Journal of Zoology*, 33, 655–665.

Nagy, K.A. & Milton, K. (1979) Energy metabolism and food consumption by wild howler monkeys (*Alouatta palliata*), *Ecology*, 60, 475–480.

Nagy, K.A. & Montgomery, G.G. (1980) Field metabolic rate, water flux, and food consumption in 3-toed sloths (*Bradypus variegatus*), *Journal of Mammalogy*, 61, 465–472.

Nagy, K.A. & Obst, Bryan, S. (1992) Food and energy requirements of Adélie penguins (*Pygoscelis adeliae*) on the Antarctic Peninsula, *Physiological Zoology*, 65, 1271–1284.

Nagy, K.A., Siegfried, W.R. & Wilson, R.P. (1984) Energy utilization by free-ranging jackass penguins, *Spheniscus demersus*, *Ecology*, 65, 1648–1655.

Nagy, K.A. & Suckling, G.C. (1985) Field energetics and water balance of sugar gliders, *Petaurus breviceps*, *Australian Journal of Zoology*, 33, 683–691.

Obst, B.A., Nagy, K.A. & Ricklefs, R.E. (1987) Energy utilization by Wilson's storm-petrel (*Oceanites oceanicus*), *Physiological Zoology*, 60, 200–210.

Obst, B.S. & Nagy, K.A. (1992) Field energy expenditures of the southern Giant-Petrel, *The Condor*, 94, 801–810.

Ochocińska, D. & Taylor, J.R.E. (2005) Living at the physiological limits: Field and maximum metabolic rates of the common shrew (*Sorex araneus*), *Physiological and Biochemical Zoology*, 78, 808–818.

Peterson, R.M., Batzli, G.O. & Banks, E.M. (1976) Activity and energetics of the brown lemming in its natural habitat, *Arctic and Alpine Research*, 8, 131–138.

Piersma, T., Lindström, Ä., Drent, R.H., I, T., Jukema, J., Morrison, R.I.G., Reneerkens, J., Schekkerman, H. & Visser, G.H. (2003) High daily energy expenditure of incubating shorebirds on high Arctic tundra: a circumpolar study, *Functional Ecology*, 17, 356–362.

Piersma, T. & Morrison, R.I.G. (1994) Energy expenditure and water turnover of incubating ruddy turnstones: high costs under high Arctic climatic conditions, *The Auk*, 111, 366–376.

Pontzer, H., Raichlen, D.A., Shumaker, R.W., Ocobock, C. & Wich, S.A. (2010) Metabolic adaptation for low energy throughput in orangutans, *Proceedings of the National Academy of Sciences of the United States of America*, 107, 14048–14052.

Powers, D.R. & Conley, T.M. (1994) Field metabolic rate and food consumption of two sympatric hummingbird species in southeastern Arizona, *The Condor*, 96, 141–150.

Powers, D.R. & Nagy, K.A. (1988) Field metabolic rate and food consumption by free-living Anna's hummingbirds (*Calypte anna*), *Physiological Zoology*, 61, 500–506.

Quin, D.G., Riek, A., Green, S., Smith, A.P. & Geiser, F. (2010) Seasonally constant field metabolic rates in free-ranging sugar gliders (*Petaurus breviceps*), *Comparative Biochemistry and Physiology Part A: Molecular & Integrative Physiology*, 155, 336–340.

Randolph, J.C. (1980) Daily energy metabolism of 2 rodents (*Peromyscus leucopus* and *Tamias striatus*) in their natural environment, *Physiological Zoology*, 53, 70–81.

Riek, A., van der Sluijs, L. & Gerken, M. (2007) Measuring the energy expenditure and water flux in free-ranging alpacas (*Lama pacos*) in the Peruvian Andes using the doubly labelled water technique, *Journal of Experimental Zoology*, 307A, 667–675.

Sanz, J.J., Tinbergen, J.M., Orell, M. & Rytkönen, S. (1998) Daily energy expenditure during brood rearing of Great Tits Parus major in northern Finland, *Ardea*, 86, 101–107.

Schmid, J. & Speakman, J.R. (2000) Daily energy expenditure of the grey mouse lemur (*Microcebus murinus*): a small primate that uses torpor, *Journal of Comparative Physiology B: Biochemical, Systemic, and Environmental Physiology*, 170, 633–641.

Schmid, J. & Speakman, J.R. (2009) Torpor and energetic consequences in free-ranging grey mouse lemurs (*Microcebus murinus*): a comparison of dry and wet forests, *Naturwissenschaften*, 96, 609–620.

Schmid, J., Andersen, N.A., Speakman, J.R. & Nicol, S.C. (2003) Field energetics of free-living, lactating and non-lactating echidnas, *Comparative Biochemistry and Physiology Part A: Molecular & Integrative Physiology*, 136, 903–909.

Seymour, R.S., Withers, P.C. & Weathers, W.W. (1998) Energetics of burrowing, running and free-living in the Namib Desert golden mole (*Eremitalpa namibensis*), *Journal of Zoology*, 244, 107–117.

Shaffer, S.A., Costa, D.P. & Weimerskirch, H. (2001) Comparison of methods for evaluating energy expenditure of incubating wandering albatrosses, *Physiological and Biochemical Zoology*, 74, 823–831.

Sheriff, M.J., Speakman, J.R., Kuchel, L., Boutin, S. & Humphries, M.M. (2009) The cold shoulder: free-ranging snowshoe hares maintain a low cost of living in cold climates, *Canadian Journal of Zoology*, 87, 956–964.

Simmen, B., Bayart, F., Rasamimanana, H., Zahariev, A., Blanc, S. & Pasquet, P. (2010) Total energy expenditure and body composition in two free-living sympatric lemurs, *PLoS One*, 5, e9860.

Tatner, P. (1990) Energetic demands during brood rearing in the Wheatear *Oenanthe oenanthe*, *Ibis*, 132, 423–435.

Taylor, J.R.E., Place, A.R. & Roby, D.D. (1997) Stomach oil and reproductive energetics in Antarctic prions, *Pachyptila desolata*, *Canadian Journal of Zoology*, 75, 490–500.

Thomas, D.W., Brigham, R.M. & Lapierre, H. (1996) Metabolic rates and body mass changes in common poorwills (*Phalaenoptilus nuttallii*: Caprimulgidae), *Ecoscience*, 3, 70–74.

Thomas, D.W., Martin, K. & Lapierre, H. (1994) Doubly labelled water measurements of field metabolic rate in white-tailed ptarmigan: variation in background isotope abundances and effect on CO_2 production estimates, *Canadian Journal of Zoology*, 72, 1967–1972.

Thomson, D.L., Furness, R.W. & Monaghan, P. (1998) Field metabolic rates of kittiwakes *Rissa tridactyla* during incubation and chick rearing, *Ardea*, 86, 169–175.

Tjørve, K.M.C., Underhill, L.G. & Visser, G.H. (2006) Energetics of growth in semi-precocial shorebird chicks in a warm environment: The African black oystercatcher, *Haematopus moquini*, *Zoology*, 110, 176–188.

Tulp, I., Schekkerman, H., Bruinzeel, L.W., Jukema, J., Visser, G.H. & Piersma, T. (2009) Energetic demands during incubation and chick rearing in a uniparental and a biparental shorebird breeding in the high Arctic, *The Auk*, 129, 155–164.

Utter, J.M. & LeFebvre, Eugene, A. (1973) Daily energy expenditure of purple martins (*Progne subis*) during the breeding season: estimates using D_2O^{18} and time budget methods, *Ecology*, 54, 597–604.

Uttley, J., Tatner, P. & Monaghan, P. (1994) Measuring the daily energy expenditure of free-living arctic terns (*Sterna paradisaea*), *The Auk*, 111, 453–459.

Voigt, C.C., Kelm, Detlev, H. & Visser, G.H. (2006) Field metabolic rates of phytophagous bats: do pollination strategies of plants make life of nectar-feeders spin faster?, *Comparative Biochemistry and Physiology Part B: Biochemistry & Molecular Biology*, 176, 213–222.

Wallis, I.R. & Green, B. (1992) Seasonal field energetics of the rufous rat-kangaroo (*Aepyprymnus rufescens*), *Australian Journal of Zoology*, 40, 279–290.

Weathers, W.W., Hodum, P.J. & Blakesley, J.A. (2001) Thermal ecology and ecological energetics of California spotted owls, *The Condor*, 103, 678–690.

Weathers, W.W., Koenig, W.D. & Stanback, M.T. (1990) Breeding energetics and thermal ecology of the acorn woodpecker in central coastal California, *The Condor*, 92, 341–359.

Weathers, W.W. & Nagy, K.A. (1980) Simultaneous doubly labeled water ($^3HH^{18}O$) and time budget estimates of daily energy-expenditure in *Phainopepla nitens*, *The Auk*, 97, 861–867.

Weathers, W.W. & Nagy, K.A. (1984) Daily energy expenditure and water flux in black-rumped waxbills (*Estrilda troglodytes*), *Comparative Biochemistry and Physiology Part A: Molecular & Integrative Physiology*, 77, 453–458.

Weathers, W.W. & Paton, D.C. (1997) Summer field metabolic rate and water intake rate in superb fairy-wrens and a white-throated treecreeper, *Emu*, 97, 324–325.

Weathers, W.W., Paton, D.C. & Seymour, R.S. (1996) Field metabolic rate and water flux of nectarivorous honeyeaters, *Australian Journal of Zoology*, 44, 445–460.

Weathers, W.W. & Stiles, G.F. (1989) Energetics and water balance in free-living tropical hummingbirds, *The Condor*, 91, 324–331.

Westerterp, K.R. & Bryant, D.M. (1984) Energetics of free existence in swallows and martins (Hirundinidae) during breeding: a comparative study using doubly labeled water, *Oecologia*, 62, 376–381.

Williams, J.B., Anderson, M.D. & Richardson, P.R.K. (1997) Seasonal differences in field metabolism, water requirements, and foraging behaviour of free-living aardwolves, *Ecology*, 78, 2588–2602.

Williams, J.B., Bradshaw, D. & Schmidt, L. (1995) Field metabolism and water requirements of spinifex pigeons (*Geophaps plumifera*) in western Australia, *Australian Journal of Zoology*, 43, 1–15.

Williams, J.B., Lenain, D., Ostrowski, S., Tieleman, B.I. & Seddon, P.J. (2002) Energy Expenditure and Water Flux of Rüppell's Foxes in Saudi Arabia, *Physiological and Biochemical Zoology*, 75, 479–488.

Williams, J.B., Ostrowski, S., Bedin, E. & Ismail, K. (2001) Seasonal variation in energy expenditure, water flux and food consumption of Arabian oryx *Oryx leucoryx*, *The Journal of Experimental Biology*, 204, 2301–2311.

Williams, J.B., Seigfried, W.R., Milton, Suzanne, J., Adams, N.T., Dean, W.R.J., du Plessis, M.A. & Jackson, S. (1993) Field metabolism, water requirements, and foraging behavior of wild ostriches in the Namib, *Ecology*, 74, 390–404.

Williams, J.B., Withers, P.C., Bradshaw, S.D. & Nagy, K.A. (1991) Metabolism and Water Flux of Captive and Free-Living Australian Parrots, *Australian Journal of Zoology*, 39, 131–142.

Williams, J.B. (1987) Field metabolism and food consumption of savannah sparrows during the breeding season, *The Auk*, 104, 277–289.

Williams, J.B. (1988) Field metabolism of tree swallows during the breeding season, *The Auk*, 105, 706–714.

Williams, J.B. (1993) Energetics of incubation in free-living orange-breasted sunbirds in South Africa, *The Condor*, 95, 115–126.

Williams, J.B. & Dwinnel, B. (1990) Field metabolism of free-living female savannah sparrows during incubation: a study using doubly labeled water, *Physiological Zoology*, 63, 353–372.

Williams, J.B. & Nagy, K.A. (1984) Daily energy expenditure of savannah sparrows: comparison of time-energy budget and doubly-labeled water estimates, *The Auk*, 101, 221–229.

Williams, J.B. & Nagy, K.A. (1985) Daily energy expenditure by female savannah sparrows feeding nestlings, *The Auk*, 102, 187–190.

Williams, J.B. & du Plessis, M.A. (1996) Field metabolism and water flux of sociable weaver *Philetairus socius* in the Kalahari Desert, *Ibis*, 138, 168–171.

Winstanley, R.K., Buttemer, W.A. & Saunders, G. (2003) Field metabolic rate and body water turnover of the red fox *Vulpes vulpes* in Australia, *Mammal Review*, 33, 295–301.
